# Supplementary material for: Peer-driven intervention to help patients resume CPAP therapy following discontinuation: a multicentre, randomised clinical trial with patient involvement
Source: BMJ Open. 2021 Oct 14;11(10):e053996. doi: 10.1136/bmjopen-2021-053996 (PMC8522667; doi:10.1136/bmjopen-2021-053996)
Supplement: Author's manuscript [file bmjopen-2021-053996.draft_revisions.pdf]

BMJ Open is committed to open peer review. As part of this commitment we make the peer review history of every article we publish publicly available.

When an article is published we post the peer reviewers' comments and the authors' responses online. We also post the versions of the paper that were used during peer review. These are the versions that the peer review comments apply to.

The versions of the paper that follow are the versions that were submitted during the peer review process. They are not the versions of record or the final published versions. They should not be cited or distributed as the published version of this manuscript.

BMJ Open is an open access journal and the full, final, typeset and author-corrected version of record of the manuscript is available on our site with no access controls, subscription charges or pay-per-view fees (http://bmjopen.bmj.com).

If you have any questions on BMJ Open's open peer review process please email info.bmjopen@bmj.com

BMJ Open

A peer-driven intervention to help patients resume CPAP therapy following discontinuation: a multicenter, randomized clinical trial with patient involvement

Journal:	*BMJ Open*
Manuscript ID	bmjopen-2021-053996
Article Type:	Protocol
Date Submitted by the Author:	31-May-2021
Complete List of Authors:	Merle, Raymond<br>Pison, Christophe; Centre Hospitalier Universitaire Grenoble Alpes, ☐ Service Hospitalier Universitaire Pneumologie Physiologie, Pôle Thorax et Vaisseaux<br>Logerot, Sophie; Agir à dom<br>Deschaux, Christelle; AGIR A DOM,<br>Arnol, Nathalie<br>Roustit, Matthieu; Centre Hospitalier Universitaire de Grenoble,<br>Tamisier, Renaud; Univ. Grenoble Alpes, HP2; Inserm, U1042; Grenoble Alps University Hospital, EFCR Laboratory<br>Pepin, Jean; Hôpital Universitaire de Grenoble,<br>BOREL, Jean; Centre Hospitalier Universitaire Grenoble Alpes
Keywords:	RESPIRATORY MEDICINE (see Thoracic Medicine), SLEEP MEDICINE, Chronic airways disease < THORACIC MEDICINE

SCHOLARONE™
Manuscripts

I, the Submitting Author has the right to grant and does grant on behalf of all authors of the Work (as defined in the below author licence), an exclusive licence and/or a non-exclusive licence for contributions from authors who are: i) UK Crown employees; ii) where BMJ has agreed a CC-BY licence shall apply, and/or iii) in accordance with the terms applicable for US Federal Government officers or employees acting as part of their official duties; on a worldwide, perpetual, irrevocable, royalty-free basis to BMJ Publishing Group Ltd ("BMJ") its licensees and where the relevant Journal is co-owned by BMJ to the co-owners of the Journal, to publish the Work in this journal and any other BMJ products and to exploit all rights, as set out in our licence.

The Submitting Author accepts and understands that any supply made under these terms is made by BMJ to the Submitting Author unless you are acting as an employee on behalf of your employer or a postgraduate student of an affiliated institution which is paying any applicable article publishing charge ("APC") for Open Access articles. Where the Submitting Author wishes to make the Work available on an Open Access basis (and intends to pay the relevant APC), the terms of reuse of such Open Access shall be governed by a Creative Commons licence – details of these licences and which Creative Commons licence will apply to this Work are set out in our licence referred to above.

Other than as permitted in any relevant BMJ Author's Self Archiving Policies, I confirm this Work has not been accepted for publication elsewhere, is not being considered for publication elsewhere and does not duplicate material already published. I confirm all authors consent to publication of this Work and authorise the granting of this licence.

For peer review only

31-05-2021 version, *BMJ open Protocol*

A peer-driven intervention to help patients resume CPAP therapy following discontinuation: a multicenter, randomized clinical trial with patient involvement

Raymond Merle[1,2,3], Christophe Pison[1-4], Sophie Logerot[5,6], Chrystèle Deschaux[5,6], Nathalie Arnol[5,6], Matthieu Roustit[1,7,8], Renaud Tamiser[1,8,9], Jean Louis Pepin[1,4,8*], Jean Christian Borel[5,6,8*]

**co-seniors authors JLP, JCB*

1- Université Grenoble Alpes, France
2- Département Universitaire des Patients Grenoble Alpes, Université Grenoble Alpes, France
3- Laboratoire de Bioénergétique Fondamentale et Appliquée, LBFA, Inserm1055, Saint Martin d'Hères, France
4- Service Hospitalier Universitaire Pneumologie, Pôle Thorax et Vaisseaux, CHU Grenoble Alpes, France
5- *AGIR* à dom. Home assistance and services, Meylan, France
6- *IC@*dom., Investigation Clinique à domicile, Meylan, France
7- CIC Inserm, CHUGA
8- Laboratoire Hypoxie et PhysioPathologies cardiovasculaires et respiratoires, HP2, Inserm1042, Grenoble, France

Corresponding author: Pr. Christophe Pison, Service Hospitalier Universitaire Pneumologie Physiologie, Pôle Thorax et Vaisseaux, CHU Grenoble Alpes, France, CS10217, 38043 Grenoble Cedex 9, France. Phone + 33 6 83 31 97 81, cpison@chu-grenoble.fr

For peer review only - http://bmjopen.bmj.com/site/about/guidelines.xhtml

31-05-2021 version, *BMJ open Protocol*

ABSTRACT

Introduction

Obstructive Sleep Apnoea Syndrome (OSAS) is one of the most common chronic diseases. It may be associated with symptoms of excessive daytime sleepiness and neurocognitive and cardiovascular complications. First line therapy for OSAS involves home Continuous Positive Airway Pressure (CPAP), however nearly half of patients do not adhere with this treatment over the long-term. Cognitive-behavioural interventions that include health professionals and patient and public involvement (PPI) are increasingly advocated in the fields of education and research. We hypothesize that a peer-driven intervention could help patients with OSAS to resume CPAP use after discontinuation.

Methods and analysis

We have designed a prospective, multicentre randomized, controlled trial that will be co-conducted by health professionals, a home provider of CPAP and patients as experts or peers or participants. The primary aim is to evaluate the impact of a 6-month, peer-driven intervention to promote the resumption of CPAP after discontinuation. We anticipate that 20% of patients in the intervention group will reuse CPAP as compared to 6% in control group, thus 104 patients must be included in each group. The secondary aims are i) to evaluate the impact of the peer-driven intervention on adherence to CPAP compared to the control group (mean adherence and percentage of nights with at least 4 hours' use /night for 70% of nights); - ii) to determine factors associated with resumption of CPAP; -iii) to assess patient satisfaction with the peer-driven intervention at 6 months; -iv) to evaluate the feasibility and the execution of the peer-driven intervention and peer satisfaction. Adult outpatients with an established diagnosis of severe OSA (Apnea-Hypopnea Index >30 events/hour) that have stopped using CPAP within 4 to 12 months after initiation will be recruited. The peers who will perform the intervention will be patients with OSAS treated with CPAP with good adherence (at least 4 hours/night, 70% of nights) and trained in

For peer review only

motivational enhancement and cognitive-behavioural therapies. Trained peers will conduct 3 interviews within 6 months with participants.

Ethics and dissemination

Ethical approval has been obtained from the French Regional Ethics Committee CPP Ouest II-Angers, (IRB 21.02.25.68606 (2021/25)). All participants will sign written informed consent. The results will be presented at conferences and published in peer-reviewed journals as well as public media.

Trial registration number: NCT04538274

Strengths and limitations of this study

- Patient involvement (PI) from the beginning of the setup of this trial. RM, the first author, is a patient expert who has completed a PhD devoted to the roles of patients in the health care system.
- The need to help patients to resume CPAP after discontinuation is currently unmet. There is a robust rationale supporting the use of motivational enhancement and cognitive-behavioural therapies performed by peers to promote CPAP resumption.
- Patient-peers with OSAS who are compliant with CPAP are probably the best stakeholders to help non-compliant patients to resume CPAP.
- Our team has experience in patient and public involvement (PPI) from work undertaken in the Grenoble Alpes University Hospital and the Grenoble Alpes University Department of Patients.

Key words: patient and public involvement (PPI), obstructive sleep apnoea syndrome (OSAS), excessive daytime sleepiness, non-adherence, motivational enhancement and cognitive-behavioural therapies

For peer review only - http://bmjopen.bmj.com/site/about/guidelines.xhtml

For peer review only

Abbreviations and website addresses

AGIR à dom.	Home care and services, Meylan, France, https://www.agiradom.com/en/
AHI	Apnoea + Hypopnea Index
CPAP	Continuous Positive Airway Pressure
DUPGA	Département Universitaire des Patients Grenoble Alpes: Grenoble Alpes University Department of Patients, DUPGA@univ-grenoble-alpes.fr
EDS	Excessive Daytime Sleepiness
OSAS	Obstructive Sleep Apnoea Syndrome
PI	Patient Involvement
PPI	Patient and Public Involvement

For peer review only

For peer review only - http://bmjopen.bmj.com/site/about/guidelines.xhtml

INTRODUCTION

Obstructive Sleep Apnoea syndrome (OSAS) is one of the most common chronic diseases. It is characterized by recurrent episodes of upper airway collapse during sleep, and may or may not be associated symptoms of excessive daytime sleepiness (EDS) and neurocognitive and cardiovascular complications [1]. Twelve million adults aged between 30 and 69 years may have moderate to severe OSAS in France, based on an Apnoea Hypopnea Index (AHI) threshold value of 15 or more events per hour of sleep [2]. The risks associated with the disease can be severe, for example, individuals with untreated OSAS have a three times greater risk of motor vehicle accidents than the general population [3]. OSAS is also associated with an increased risk of cardiovascular disease, diabetes and glucose dysregulation [4], independent from obesity [5].

The first line therapy for OSAS is continuous positive airway pressure (CPAP) [1,6,7]. CPAP has been shown to effectively reduce EDS and to improve daily functioning, cognitive function, mood and quality of life [3,6]. The use of CPAP also reduces traffic accidents [7] and other work-related injuries, and improves work productivity [8]. Although CPAP therapies are highly effective in normalizing AHI and reducing symptoms in symptomatic patients, treatment success is limited by long term nonadherence in nearly half of patients [9]. Technical progress in the systems and interfaces (soundproofing, improved masks, humidification, pressure modulation, etc.) have unfortunately not been sufficient to improve compliance [10,11]. Equally, the effect sizes of telemedicine approaches are not as large as what has been achieved with the use of behavioural therapies, and the impacts on patient and provider satisfaction and cost-effectiveness are not yet clear [12–15].

Nonadherence is related to users' profiles, their representations of OSAS and the benefits they experience from CPAP [12,16,17]. This is why cognitive-behavioural and motivation enhancement therapies conducted by health professionals could be effective in

For peer review only - http://bmjopen.bmj.com/site/about/guidelines.xhtml

ensuring adherence to CPAP. A Cochrane review in 2014 showed that there is a low level of evidence that such interventions increase CPAP use (by 1.44 h per night in six studies; n = 584) and increase the number of participants who used their devices for longer than four hours per night (from 28 to 47% in 3 studies; n= 358)[18]. More robust studies are thus needed to increase the level of evidence regarding these types of interventions.

In addition, patient and public involvement (PPI) is more and more advocated in the fields of health education and research [19–25].Nevertheless, the efficacy of PPI remains to be demonstrated [26]. To our knowledge, only one previous pilot study in 39 patients showed that one-to-one peer support at CPAP initiation was feasible and generated high patient satisfaction. However, the study was not powerful enough to demonstrate effectiveness in terms of adherence to CPAP [18,27]. The data from the study, are, however, useful for designing further studies.

The aim of this adequately powered randomized clinical trial is therefore to assess the role of trained Patient Involvement (PI) representatives to help patients with OSAS to restart CPAP after discontinuation.

METHODS AND ANALYSIS

Study design

This is a prospective, multicentre, randomized controlled trial that will be co-conducted by health professionals, a CPAP home provider and patients as experts or peers or participants. After signing a consent form, patients' participants will be randomized 1.1 to the intervention group with peers or the control group. *Nota bene:* the peers involved in the conduct of the study will sign a confidentiality agreement of non-divulgation of the information exchanged with the participants.

Objectives

Primary research aim

For peer review only - http://bmjopen.bmj.com/site/about/guidelines.xhtml

The primary aim is to evaluate the impact of a 6-month intervention involving trained PI representatives to promote the resumption of CPAP in patients who have discontinued its use. Resumption of CPAP is defined as the medical prescription and the setting up of a new CPAP device at home by the homecare provider.

Secondary research aims:

i) to evaluate the impact of the peer-driven intervention on adherence to CPAP by comparing adherence with the control group (mean adherence and % of nights with at least 4 hours' use /night for 70 % of nights);

ii) to determine the factors associated with the resumption of CPAP treatment;

iii) to assess the satisfaction of the intervention group with the peer-driven intervention at 6 months;

iv) to evaluate the feasibility and the execution of the peer-driven intervention and the satisfaction of peers after the interviews conducted.

Patients, Table 1

Adults with an established diagnosis of severe OSAS (AHI >30 events/hour) who have discontinued CPAP by returning their device to the homecare provider within 4 to 12 months after CPAP initiation will be recruited according to the study flow chart depicted in Figure 1.

Interventions (Figure 1)

Recruitment and training of PI representatives

PI representatives will be recruited from the investigators clinics. To be recruited as a PI representative, patients should:

- have used home CPAP for at least one year,
- have a CPAP adherence of at least 4 h/night for 70% of nights,

For peer review only - http://bmjopen.bmj.com/site/about/guidelines.xhtml

- express their motivation in participating in a training and orientation session conducted by research staff and including expert patients from the Grenoble Alpes University Department of Patients (DUP GA) [28],
- accept to conduct 3 motivational sessions by videoconference meetings of 45 to 60 minutes duration with 5 to 8 patients within 6 months after each patient's inclusion,

Patients with any major psychiatric illness, shift-workers or frequent out of town travellers will not be recruited as peers.

Peers will be trained during a three half-days interactive session organised by DUP GA, with experts in patient therapeutic education and communication, and investigators [28]. Peers will be taught how to interact with the patients recruited in the study: the aim is for them to share their experiences but not to provide any medical advice.

Description of the intervention

Trained peers will meet patients randomized into the intervention group by videoconference. Each PI representative will be allocated 5 to 8 patients. They will conduct 3 face to face motivational sessions, each of 45 to 60 minutes duration, over a 6-month period based on the principle of motivational enhancement and cognitive-behavioural therapies [11,13]. The content of the first session is designed to identify and understand the underlying reasons for stopping CPAP treatment and to identify difficulties encountered by the patient (advantages and disadvantages of CPAP treatment). The aim of the second session will be for the patient to define his/her objectives and priorities. During the last session, will be discussed to strengthen the motivation to change and how to plan for it. The peers will receive 100 € per patient for the 3 interviews.

In the control group, patients will be informed, at inclusion, that they can have a visit with a physician investigator at any time to resume treatment if they wish, as is usual practice. At the end of the six-month follow-up period, all patients in both groups will have a consultation with their physician who will suggest they resume CPAP treatment. This visit

For peer review only - http://bmjopen.bmj.com/site/about/guidelines.xhtml

may take place earlier if the patient wishes to resume CPAP treatment before the end of the follow-up period.

Assessment

Average adherence to CPAP will be measured from data recorded by the built-in software of the CPAP devices (via tele monitoring or retrieved by a home technician) for 1 month after the final consultation.

The relationship between the variables below and a positive response to the peers intervention (defined by a restart of CPAP treatment) will be analyzed: age, sex, Body Mass Index (BMI), marital status, education level, socio-professional status, precariousness (using the EPICES score), smoking and alcohol use, comorbidities (using Charlson score), history of OSAS (date of diagnosis of OSAS, baseline AHI), observance to treatments (Girerd score), date and reason for stopping CPAP and EDS score (using the Epworth Sleepiness Scale).

To determine patient profiles, their representations of OSAS, their experiences with CPAP and their knowledge and confidence to manage their health, 3 questionnaires will be completed at inclusion (M0) and at the 6-monthfollow-up (M6): the Functional Outcomes of Sleep Questionnaire (FOSQ) a disease-specific quality of life questionnaire [29], the Patient Activation Measure (PAM) a measure that assesses patient knowledge, skill, and confidence for self-management [30] and the Self-Efficacy Measure for Sleep Apnea (SEMSA) [31,32] a tool with strong psychometric properties that identifies patient perceptions that may indicate those most likely not to adhere to treatment.

The satisfaction of participating patients with the PI intervention and the satisfaction of PI representatives will be measured on a 4-point Likert scale: very dissatisfied, dissatisfied, satisfied, very satisfied.

Finally, the feasibility and the execution of the 3 interviews will be assessed by the number of interviews carried out in their entirety and the average duration of each

For peer review only - http://bmjopen.bmj.com/site/about/guidelines.xhtml

interview (in minutes).

All information will be collected in secure electronic medical records in accordance with the requirements of General Data Protection Regulation.

Statistical analysis

Sample size

We hypothesize that 20% of patients allocated to the intervention group will reuse CPAP 6 months as compared to 6% of patients in the control group. A two group χ^2 test with a 5% two-sided significance level will have 80% power to detect such difference between the two groups when the sample size in each group is 90 (nQuery v8, Statistical Solutions, Cork, Ireland). In order to take into account a possible drop-outs and to comply with the intent-to-treat principle, we will inflate the sample size by a factor of 15% [33]. We thus plan to include 104 patients per group (i.e. 208 patients in total). 15 patient peers will be involved.

Feasibility and recruitment

The home care provider, *AGIR* à dom. follows more than 20,000 patients with OSAS who use CPAP in the south of France. In 2018, out of 3,281 patients who started CPAP within the study area (Isère, Savoie and Haute-Savoie), 365 discontinued it between 4 to 12 months post initiation and 6% resumed use within 6 months after discontinuation.

Randomization

After consent, randomization will be performed by a centralized computer software for each investigating center. It will be stratified on the center.

Statistical analysis plan

Descriptive analyses: continuous variables will be expressed as medians (25th/75th percentiles) or means (SD) depending on normality which will be assessed with the Shapiro-Wilk test. Categorical variables will be reported as absolute numbers and percentages for both groups. Baseline comparisons between groups will be made using a

For peer review only - http://bmjopen.bmj.com/site/about/guidelines.xhtml

Student's t-test or Mann-Whitney U test, depending on the distribution. For discrete variables, a $\chi 2$ test will be used. If significant differences are observed between arms, ANOVA and multivariable regression will be performed. In the case of missing data, an imputation strategy will be applied according to the percentage of missing values. Data management and statistical analyses will be performed using SAS, V.9.4, SAS Institute.

Primary outcome analysis: the impact of the PI intervention on the resumption of CPAP treatment will be studied by comparing the resumption of CPAP in the 2 arms, using a Chi-square test. To take into account a possible centre effect, a second analysis will be carried out using a conditional logistic regression stratified by the centre; the intervention or control arm will be considered as the dependent variable.

Secondary outcomes analyses: mean CPAP compliance one month after resumption of CPAP will be analysed using a mixed linear model (fixed factor: randomisation arm (intervention vs. control), random factor: centre). Comparison of the probability of resuming CPAP with an average compliance of at least 4 hours/night, 70% of nights between the intervention and control groups will be analysed using a conditional logistic regression, stratified by centre. All analyses will be performed as intention-to-treat and then a sensitivity analysis will also be performed *per protocol* (patients who have not resumed treatment will be considered to have zero adherence).

The association between resumption of CPAP and the sociodemographic parameters, clinical data and the scores of the three questionnaires will be studied by conditional logistic regression models stratified by centre, and adjusted by arm (intervention vs control).

In the intervention arm, descriptive statistics will be presented on the satisfaction as well as on the number of interviews carried out and their average duration.

Ethics

For peer review only - http://bmjopen.bmj.com/site/about/guidelines.xhtml

31-05-2021 version, *BMJ open Protocol*

The study will be conducted in accordance with the Declaration of Helsinki and the recommendations for Good Clinical Practice. Written informed consent will be signed by all study participants before enrolment in the study. Patients will have the right to withdraw from the study without incurring any prejudice at any time.

Patient involvement

RM, first author and expert patient, and members of DUP GA participated in the design of this study and will participate in all stages including teaching peers [28] and promoting and reporting the data, including publication in peer review. Thanks to training with health professionals and expert patients [22,23,25] peers will adopt the appropriate posture to enable patients to find their own resources to overcome barriers to use CPAP.

Dissemination

Dissemination plans of the results include presentations at conferences and a publication in peer-reviewed journal. Updates of the randomized trial will be available at ClinicalTrials.com. All patients will be informed that the dissemination of results will be accessible on request.

Sponsor and funding

The study sponsor will be AGIR à dom. Co-Principal investigators are RM, an expert patient, and JCB, a researcher. The collaborators and sponsors were not involved in the design of the study and will not influence the execution, analysis or publication of results.

DISCUSSION

OSAS is associated with many negative health consequences [1]. The lack of compliance with home CPAP therapy, which is the first line of treatment, and which has shown to be effective on quality of life is a major issue both in terms of the patient's own health status and in health care utilisation [1,2,7,8]. Attempts have been made to improve CPAP compliance by improving technical issues relating to the comfort of use of the system

For peer review only - http://bmjopen.bmj.com/site/about/guidelines.xhtml

[10,11] and the use of the of remote monitoring and telemedicine, along with the implementation of web-based adherence interventions [12–15]; however they have not been shown to improve compliance with the therapy. Other strategies to improve compliance therefore need to be developed and tested.

One of the main strengths of this study is the involvement of peers in the implementation of the behavioural intervention. Regarding efficacy, the involvement of patients with experience in the motivation of their peers to comply with treatment has been implemented with success in other chronic conditions requiring self-management such as HIV and diabetes [34,35]. Furthermore, evidence suggests that patients perceive peers with similar comorbidities as more credible than health-care professionals in the delivery of behavioural interventions [36–38]. The concept of PPI in education and research has been adopted by a growing number of medical schools, particularly in the United kingdom [19,24]. If the results of this study confirm the effectiveness of the PI intervention in promoting resumption of CPAP in patients initially failing CPAP, this study will provide an evidence base to support the use of PI in the management of OSAS in conjunction with the home healthcare provider and specialized sleep centers [39].

The aim to seek factors that are related to CPAP resumption will provide useful information regarding those patients who are more likely to resume CPAP and therefore who PI interventions are more likely to help. This will open the way for further studies to determine the most appropriate methods to improve compliance in those patients who benefit less from PI interventions.

Despite these strengths, the study has two main inherent limitations. Firstly, the results are likely to be biased by the fact that patients who accept to participate may be more likely to resume CPAP therapy than those who decline participation. The results may therefore not be generalizable to all patients who have stopped using their CPAP as prescribed. Secondly, the effectiveness of the intervention may also depend on the capacity of the peer-

For peer review only - http://bmjopen.bmj.com/site/about/guidelines.xhtml

participant to deliver it. The training is quite short (3 half-days) and some of the peers recruited may be more skilled than others in providing such intervention. However, in this study, the peers will be additionally supported throughout the study by the University Department of Patients.

In summary, the results of this study will determine the effectiveness of a PI intervention to motivate patients who have stopped using their CPAP as prescribed to resume its use on compliance with CPAP therapy. The results will also provide information regarding the factors relating to resumption of CPAP, providing a starting point for further studies to determine the most appropriate methods to improve compliance in those patients who benefit less from PI interventions.

For peer review only

For peer review only - http://bmjopen.bmj.com/site/about/guidelines.xhtml

Acknowledgements We specially thank John Louis McGregor, PhD, retired Director of Medical Research (DR1) at INSERM, former director of INSERM Unit 331, and retired Honorary Senior Lecturer (research) at the Cardiovascular division King's College London, for his continuous encouragements and counselling together with manuscript reviewing.

Contributors RM participated in the design of the study, wrote the article based on the study protocol, will train PI, collect and analyse data into the protocol. CP participated in the design of the study, wrote the study protocol and will include patients into the protocol together with PPI. SL participated in the design of the study, wrote the study protocol. CD and NA participated in establishing the sample size and will help to recruit patients. MR set up statistical analysis plan and determine sample size. RT revised the manuscript, will include patients into the protocol and collect and analyse data. JLP designed the study, critically revised the manuscript, will include patients, and collect and analyse data. JCB designed the study, critically revised the manuscript and will analyse data. The submitted manuscript has been approved by all authors.

Funding Mr. R. Merle is a recipient of a grant from Agir pour les Maladies Chroniques, http://fonds-apmc.org received in 2019. JLP and RT are supported by the French National Research Agency in the framework of the "Investissements d'avenir" program (ANR-15-IDEX-02) and the "e-health and integrated care and trajectories medicine and MIAI artificial intelligence" Chairs of excellence from the Grenoble-Alpes University Foundation. This work has been partially supported by MIAI @ Grenoble Alpes, (ANR-19-P3IA-0003).

Competing interests Mr. R. Merle is a recipient of a grant from Agir pour les Maladies

For peer review only

Chroniques, http://fonds-apmc.org/. CD, NA, JCB are employees of *AGIR* à dom. CP and JLP received grants from Agir pour les Maladies Chroniques, http://fonds-apmc.org/.

Ethics approval

The protocol to be approved by The French Regional Ethics Committee CPP Ouest II-Angers.

Provenance and peer review: not commissioned; externally peer reviewed.

Open access This is an open access article distributed in accordance with the Creative Commons Attribution Non Commercial (CC BY-NC 4.0) license, which permits others to distribute, remix, adapt, build upon this work non-commercially, and license their derivative works on different terms, provided the original work is properly cited, appropriate credit is given, any changes made indicated, and the use is non-commercial. See: http:// creativecommons. org/ licenses/ by- nc/ 4.0/.

For peer review only

31-05-2021 version, *BMJ open Protocol*

REFERENCES

1 Lévy P, Kohler M, McNicholas WT, *et al.* Obstructive sleep apnoea syndrome. *Nat Rev Dis Primer* 2015;**1**:15015. doi:10.1038/nrdp.2015.15

2 Benjafield AV, Ayas NT, Eastwood PR, *et al.* Estimation of the global prevalence and burden of obstructive sleep apnoea: a literature-based analysis. *Lancet Respir Med* 2019;**7**:687–98. doi:10.1016/S2213-2600(19)30198-5

3 Léger D, Stepnowsky C. The economic and societal burden of excessive daytime sleepiness in patients with obstructive sleep apnea. *Sleep Med Rev* 2020;**51**:101275. doi:10.1016/j.smrv.2020.101275

4 Ryan S, Cummins EP, Farre R, *et al.* Understanding the pathophysiological mechanisms of cardiometabolic complications in obstructive sleep apnoea: towards personalised treatment approaches. *Eur Respir J* 2020;**56**. doi:10.1183/13993003.02295-2019

5 Ryan S, Arnaud C, Fitzpatrick SF, *et al.* Adipose tissue as a key player in obstructive sleep apnoea. *Eur Respir Rev Off J Eur Respir Soc* 2019;**28**. doi:10.1183/16000617.0006-2019

6 Wimms AJ, Kelly JL, Turnbull CD, *et al.* Continuous positive airway pressure versus standard care for the treatment of people with mild obstructive sleep apnoea (MERGE): a multicentre, randomised controlled trial. *Lancet Respir Med* 2020;**8**:349–58. doi:10.1016/S2213-2600(19)30402-3

7 George CF. Reduction in motor vehicle collisions following treatment of sleep apnoea with nasal CPAP. *Thorax* 2001;**56**:508–12. doi:10.1136/thorax.56.7.508

For peer review only

8 Stepnowsky C, Sarmiento KF, Bujanover S, *et al.* Comorbidities, Health-Related Quality of Life, and Work Productivity Among People With Obstructive Sleep Apnea With Excessive Sleepiness: Findings From the 2016 US National Health and Wellness Survey. *J Clin Sleep Med JCSM Off Publ Am Acad Sleep Med* 2019;**15**:235–43. doi:10.5664/jcsm.7624

9 Weaver TE, Grunstein RR. Adherence to continuous positive airway pressure therapy: the challenge to effective treatment. *Proc Am Thorac Soc* 2008;**5**:173–8. doi:10.1513/pats.200708-119MG

10 Donovan LM, Boeder S, Malhotra A, *et al.* New developments in the use of positive airway pressure for obstructive sleep apnea. *J Thorac Dis* 2015;**7**:1323–42. doi:10.3978/j.issn.2072-1439.2015.07.30

11 Rotenberg BW, Murariu D, Pang KP. Trends in CPAP adherence over twenty years of data collection: a flattened curve. *J Otolaryngol - Head Neck Surg* 2016;**45**. doi:10.1186/s40463-016-0156-0

12 Bakker JP, Weaver TE, Parthasarathy S, *et al.* Adherence to CPAP: What Should We Be Aiming For, and How Can We Get There? *Chest* 2019;**155**:1272–87. doi:10.1016/j.chest.2019.01.012

13 Hwang D, Chang JW, Benjafield AV, *et al.* Effect of Telemedicine Education and Telemonitoring on Continuous Positive Airway Pressure Adherence. The Tele-OSA Randomized Trial. *Am J Respir Crit Care Med* 2018;**197**:117–26. doi:10.1164/rccm.201703-0582OC

14 Tamisier R, Treptow E, Joyeux-Faure M, *et al.* Impact of a multimodal telemonitoring intervention on CPAP adherence in symptomatic low-cardiovascular

For peer review only

risk sleep apnea: a randomized controlled trial. *Chest* Published Online First: 5 July 2020. doi:10.1016/j.chest.2020.05.613

15 Pépin J-L, Jullian-Desayes I, Sapène M, *et al.* Multimodal Remote Monitoring of High Cardiovascular Risk Patients With OSA Initiating CPAP: A Randomized Trial. *Chest* 2019;**155**:730–9. doi:10.1016/j.chest.2018.11.007

16 Weaver TE. Novel Aspects of CPAP Treatment and Interventions to Improve CPAP Adherence. *J Clin Med* 2019;**8**. doi:10.3390/jcm8122220

17 Mendelson M, Gentina T, Gentina E, *et al.* Multidimensional Evaluation of Continuous Positive Airway Pressure (CPAP) Treatment for Sleep Apnea in Different Clusters of Couples. *J Clin Med* 2020;**9**. doi:10.3390/jcm9061658

18 Wozniak DR, Lasserson TJ, Smith I. Educational, supportive and behavioural interventions to improve usage of continuous positive airway pressure machines in adults with obstructive sleep apnoea. *Cochrane Database Syst Rev* Published Online First: 8 January 2014. doi:10.1002/14651858.CD007736.pub2

19 Spencer J, Godolphin W, Karpenko N. *Can patients be teachers? Involving patients and service users in healthcare professionals' education.* https://www.health.org.uk/sites/default/files/CanPatientsBeTeachers.pdf

20 Ocloo J, Matthews R. From tokenism to empowerment: progressing patient and public involvement in healthcare improvement. *BMJ Qual Saf*;**25**:626–32. doi:10.1136/bmjqs-2015-004839

21 Harrison JD, Auerbach AD, Anderson W. Patient stakeholder engagement in research: A narrative review to describe foundational principles and best practice

For peer review only

31-05-2021 version, *BMJ open Protocol*

activities. *Health Expect Int J Public Particip Health Care Health Policy*;**22**:307–16. doi:10.1111/hex.12873

22 Merle R, Pépin J-L, Ménissier T, *et al.* [Growing role of patients in French health democracy]. *Rev Mal Respir* 2020;**37**:620–3. doi:10.1016/j.rmr.2020.08.002

23 Merle R, Pépin J-L, Palombi O. Successful training of patient and public involvement in health education and clinical research at Grenoble Patients' University centre. *BMJ* 2020.

24 Dijk SW, Duijzer EJ, Wienold M. Role of active patient involvement in undergraduate medical education: a systematic review. *BMJ Open* 2020;**10**:e037217. doi:10.1136/bmjopen-2020-037217

25 Merle R, Casagrande A, Pariset A, *et al.* [The roles of patients in healthcare provision, training and research: A French perspective]. *Rev Epidemiol Sante Publique* Published Online First: 18 May 2021. doi:10.1016/j.respe.2021.04.136

26 Fønhus MS, Dalsbø TK, Johansen M, *et al.* Patient-mediated interventions to improve professional practice. *Cochrane Database Syst Rev* Published Online First: 2018. doi:10.1002/14651858.CD012472.pub2

27 Parthasarathy S, Wendel C, Haynes PL, *et al.* A pilot study of CPAP adherence promotion by peer buddies with sleep apnea. *J Clin Sleep Med JCSM Off Publ Am Acad Sleep Med* 2013;**9**:543–50. doi:10.5664/jcsm.2744

28 Merle R, Pépin J-L, Palombi O, *et al.* Co creation of a programme at Grenoble Patients' University to train patients to teach and participate in research. BMJ Opin. 2021.

For peer review only

31-05-2021 version, *BMJ open Protocol*

29 Weaver TE, Laizner AM, Evans LK, *et al.* An instrument to measure functional status outcomes for disorders of excessive sleepiness. *Sleep* 1997;**20**:835–43.

30 Hibbard JH, Stockard J, Mahoney ER, *et al.* Development of the Patient Activation Measure (PAM): conceptualizing and measuring activation in patients and consumers. *Health Serv Res* 2004;**39**:1005–26. doi:10.1111/j.1475-6773.2004.00269.x

31 Weaver TE, Maislin G, Dinges DF, *et al.* Self-efficacy in sleep apnea: instrument development and patient perceptions of obstructive sleep apnea risk, treatment benefit, and volition to use continuous positive airway pressure. *Sleep* 2003;**26**:727–32. doi:10.1093/sleep/26.6.727

32 Micoulaud-Franchi J-A, Coste O, Bioulac S, *et al.* A French update on the Self-Efficacy Measure for Sleep Apnea (SEMSA) to assess continuous positive airway pressure (CPAP) use. *Sleep Breath Schlaf Atm* 2019;**23**:217–26. doi:10.1007/s11325-018-1686-7

33 Ellenberg SS. Randomization Designs in Comparative Clinical Trials. *N Engl J Med* 1984;**310**:1404–8. doi:10.1056/NEJM198405243102141

34 Deering KN, Shannon K, Sinclair H, *et al.* Piloting a peer-driven intervention model to increase access and adherence to antiretroviral therapy and HIV care among street-entrenched HIV-positive women in Vancouver. *AIDS Patient Care STDs* 2009;**23**:603–9. doi:10.1089/apc.2009.0022

35 Lorig K, Ritter PL, Villa FJ, *et al.* Community-based peer-led diabetes self-management: a randomized trial. *Diabetes Educ* 2009;**35**:641–51. doi:10.1177/0145721709335006

For peer review only

31-05-2021 version, *BMJ open Protocol*

36 Heisler M, Halasyamani L, Resnicow K. I Am Not Alone": The Feasibility and Acceptability of Interactive Voice Response-Facilitated Telephone Peer Support Among Older Adults With Heart Failure. *Congest Heart Fail*;**13**:149–57. doi:10.1111/j.1527-5299.2007.06412.x

37 Solomon P. Peer support/peer provided services underlying processes, benefits, and critical ingredients. *Psychiatr Rehabil J*;**27**:392–401. doi:10.2975/27.2004.392.401

38 Gaines ME, Morris P, Thistlethwaite J. The patient ' s voice in health and social care professional education: The Vancouver Statement. *Int J Health Gov*;**21**:18–25. doi:10.1108/IJHG-01-2016-0003

39 Billings ME, Pendharkar SR. Alternative Care Pathways for Obstructive Sleep Apnea and the Impact on Positive Airway Pressure Adherence: Unraveling the Puzzle of Adherence. *Sleep Med Clin* 2020;**0**. doi:10.1016/j.jsmc.2020.10.005

For peer review only

31-05-2021 version, *BMJ open Protocol*

Table 1 -Inclusion and exclusion criteria

**Inclusion criteria**	**Exclusion criteria**
▪ Over 18 years' old<br>▪ Diagnosed with of severe OSA (AHI ≥ 30 events/hour)<br>▪ Discontinuation of CPAP 4 to 12 months after initiation and having stopped their CPAP treatment no later than one year prior to their inclusion<br>▪ Followed by the home health care provider AGIRa*Dom*<br>▪ Access to a computer and/or tablet and an internet connection<br>▪ Oral and written French<br>▪ Able to provide written informed consent<br>▪ Affiliated to social security or beneficiary of such a scheme	▪ CPAP cessation due to a resolution of the OSAS (e.g. weight loss after bariatric surgery) or another pathology that prevents the continuation of treatment (e.g. ENT surgery, etc.)<br>▪ Severe and/or unstable comorbidity that required hospitalisation for decompensation in the previous year (heart, kidney, respiratory, liver, psychiatric or other insufficiency)<br>Central sleep apnoea index above 20% of AHI at the time of diagnosis<br>▪ Patient being treated with a mandibular advancement orthosis<br>▪ Lack of availability (e.g. night worker or patient who travels frequently, etc.).<br>▪ Current participation in, participation in the month prior to inclusion in another clinical intervention research study that may impact the study: this impact is left to the investigator's discretion.<br>▪ Referred to in Articles L1121-5 to L1121-8 of the CSP (corresponds to all protected persons: pregnant woman, breastfeeding mother, person deprived of liberty by judicial or administrative decision, person subject to a legal protection measure)

For peer review only - http://bmjopen.bmj.com/site/about/guidelines.xhtml

31-05-2021 version, *BMJ open Protocol*

Figure 1, Work-flow

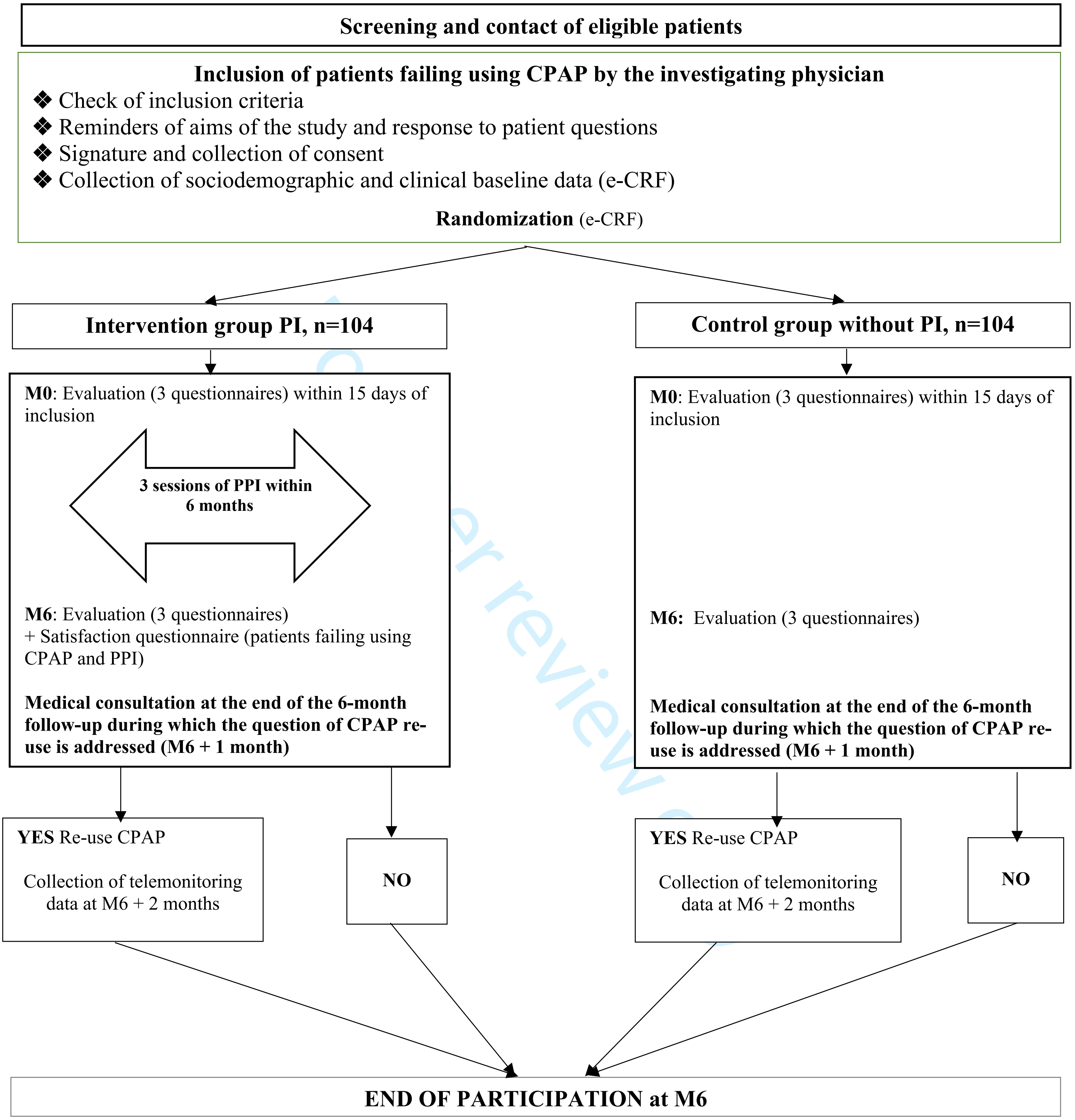

For peer review only - http://bmjopen.bmj.com/site/about/guidelines.xhtml

BMJ Open

A peer-driven intervention to help patients resume CPAP therapy following discontinuation: a multicenter, randomized clinical trial with patient involvement

Journal:	*BMJ Open*
Manuscript ID	bmjopen-2021-053996.R1
Article Type:	Protocol
Date Submitted by the Author:	07-Aug-2021
Complete List of Authors:	Merle, Raymond; Université Grenoble Alpes<br>Pison, Christophe; Centre Hospitalier Universitaire Grenoble Alpes, ☐ Service Hospitalier Universitaire Pneumologie Physiologie, Pôle Thorax et Vaisseaux; Université Grenoble Alpes<br>Logerot, Sophie; Agir à dom<br>Deschaux, Chrystèle; AGIR à Dom<br>Arnol, Nathalie; AGIR à Dom<br>Roustit, Matthieu; Centre Hospitalier Universitaire de Grenoble; Universite Grenoble Alpes<br>Tamisier, Renaud; Univ. Grenoble Alpes, HP2; Inserm, U1042; Grenoble Alps University Hospital, EFCR Laboratory<br>Pepin, Jean; Hôpital Universitaire de Grenoble; University Grenoble Alpes<br>BOREL, Jean; Centre Hospitalier Universitaire Grenoble Alpes
**Primary Subject Heading**:	Respiratory medicine
Secondary Subject Heading:	Respiratory medicine
Keywords:	RESPIRATORY MEDICINE (see Thoracic Medicine), SLEEP MEDICINE, Chronic airways disease < THORACIC MEDICINE

SCHOLARONE™
Manuscripts

I, the Submitting Author has the right to grant and does grant on behalf of all authors of the Work (as defined in the below author licence), an exclusive licence and/or a non-exclusive licence for contributions from authors who are: i) UK Crown employees; ii) where BMJ has agreed a CC-BY licence shall apply, and/or iii) in accordance with the terms applicable for US Federal Government officers or employees acting as part of their official duties; on a worldwide, perpetual, irrevocable, royalty-free basis to BMJ Publishing Group Ltd ("BMJ") its licensees and where the relevant Journal is co-owned by BMJ to the co-owners of the Journal, to publish the Work in this journal and any other BMJ products and to exploit all rights, as set out in our licence.

The Submitting Author accepts and understands that any supply made under these terms is made by BMJ to the Submitting Author unless you are acting as an employee on behalf of your employer or a postgraduate student of an affiliated institution which is paying any applicable article publishing charge ("APC") for Open Access articles. Where the Submitting Author wishes to make the Work available on an Open Access basis (and intends to pay the relevant APC), the terms of reuse of such Open Access shall be governed by a Creative Commons licence – details of these licences and which Creative Commons licence will apply to this Work are set out in our licence referred to above.

Other than as permitted in any relevant BMJ Author's Self Archiving Policies, I confirm this Work has not been accepted for publication elsewhere, is not being considered for publication elsewhere and does not duplicate material already published. I confirm all authors consent to publication of this Work and authorise the granting of this licence.

For peer review only

31-05-2021 version, *BMJ open Protocol-R1, revised document, the 4th of August 2021*

A peer-driven intervention to help patients resume CPAP therapy following discontinuation: a multicenter, randomized clinical trial with patient involvement

Raymond Merle[1,2,3], Christophe Pison[1-4], Sophie Logerot[5,6], Chrystèle Deschaux[5,6], Nathalie Arnol[5,6], Matthieu Roustit[1,7,8], Renaud Tamisier[1,8,9], Jean Louis Pepin[1,4,8*], Jean Christian Borel[5,6,8*]

**co-seniors authors JLP, JCB*

1- Université Grenoble Alpes, France
2- Département Universitaire des Patients Grenoble Alpes, Université Grenoble Alpes, France
3- Laboratoire de Bioénergétique Fondamentale et Appliquée, LBFA, Inserm1055, Saint Martin d'Hères, France
4- Service Hospitalier Universitaire Pneumologie, Pôle Thorax et Vaisseaux, CHU Grenoble Alpes, France
5- *AGIR* à dom. Home assistance and services, Meylan, France
6- *IC@*dom., Investigation Clinique à domicile, Meylan, France
7- CIC Inserm, CHUGA
8- Laboratoire Hypoxie et PhysioPathologies cardiovasculaires et respiratoires, HP2, Inserm1042, Grenoble, France

Corresponding author: Pr. Christophe Pison, Service Hospitalier Universitaire Pneumologie Physiologie, Pôle Thorax et Vaisseaux, CHU Grenoble Alpes, France, CS10217, 38043 Grenoble Cedex 9, France. Phone + 33 6 83 31 97 81, cpison@chu-grenoble.fr

For peer review only - http://bmjopen.bmj.com/site/about/guidelines.xhtml

ABSTRACT

Introduction

Obstructive Sleep Apnoea Syndrome (OSAS) is one of the most common chronic diseases. It may be associated with symptoms of excessive daytime sleepiness and neurocognitive and cardiovascular complications. First line therapy for OSAS involves home Continuous Positive Airway Pressure (CPAP), however nearly half of patients do not adhere with this treatment over the long-term. Cognitive-behavioural interventions that include health professionals and patient and public involvement (PPI) are increasingly advocated in the fields of education and research. We hypothesize that a peer-driven intervention could help patients with OSAS to resume CPAP use after discontinuation.

Methods and analysis

We have designed a prospective, multicentre randomized, controlled trial that will be co-conducted by health professionals, a home provider of CPAP and patients as experts or peers or participants. The primary aim is to evaluate the impact of a 6-month, peer-driven intervention to promote the resumption of CPAP after discontinuation. We anticipate that 20% of patients in the intervention group will reuse CPAP as compared to 6% in control group, thus 104 patients must be included in each group. The secondary aims are i) to evaluate the impact of the peer-driven intervention on adherence to CPAP compared to the control group (mean adherence and percentage of nights with at least 4 hours' use /night for 70% of nights); - ii) to determine factors associated with resumption of CPAP; -iii) to assess patient satisfaction with the peer-driven intervention at 6 months; -iv) to evaluate the feasibility and the execution of the peer-driven intervention and peer satisfaction. Adult outpatients with an established diagnosis of severe OSA (Apnea-Hypopnea Index >30 events/hour) that have stopped using CPAP within 4 to 12 months after initiation will be recruited. The peers who will perform the intervention will be patients with OSAS treated with CPAP with good adherence (at least 4 hours/night, 70% of nights) and trained in

For peer review only - http://bmjopen.bmj.com/site/about/guidelines.xhtml

motivational enhancement and cognitive-behavioural therapies. Trained peers will conduct 3 interviews within 6 months with participants.

Ethics and dissemination

Ethical approval has been obtained from the French Regional Ethics Committee CPP Ouest II-Angers, (IRB 21.02.25.68606 (2021/25)). All participants will sign written informed consent. The results will be presented at conferences and published in peer-reviewed journals as well as public media.

Trial registration number: NCT04538274

Strengths and limitations of this study

- Patient involvement (PI) from the beginning of the setup of this trial.
- There is a rationale supporting the use of motivational enhancement and cognitive-behavioural therapies performed by peers to promote CPAP resumption.
- Our team has experience in patient and public involvement (PPI) from work undertaken at the Grenoble Alpes University Department of Patients.
- Challenges are to train enough peers with homogenous skills.

Key words: patient and public involvement (PPI), obstructive sleep apnoea syndrome (OSAS), excessive daytime sleepiness, non-adherence, motivational enhancement and cognitive-behavioural therapies

Abbreviations and website addresses

AGIR à dom.	Home care and services, Meylan, France, https://www.agiradom.com/en/
AHI	Apnoea + Hypopnea Index

For peer review only

CPAP	Continuous Positive Airway Pressure
DUPGA	Département Universitaire des Patients Grenoble Alpes: Grenoble Alpes University Department of Patients, https://medecine.univ-grenoble-alpes.fr/departements/departement-universitaire-des-patients/
EDS	Excessive Daytime Sleepiness
OSAS	Obstructive Sleep Apnoea Syndrome
PI	Patient Involvement
PPI	Patient and Public Involvement

For peer review only

INTRODUCTION

Obstructive Sleep Apnoea syndrome (OSAS) is one of the most common chronic diseases. It is characterized by recurrent episodes of upper airway collapse during sleep, and may or may not be associated symptoms of excessive daytime sleepiness (EDS) and neurocognitive and cardiovascular complications [1]. Twelve million adults aged between 30 and 69 years may have moderate to severe OSAS in France, based on an Apnoea Hypopnea Index (AHI) threshold value of 15 or more events per hour of sleep [2]. The risks associated with the disease can be severe, for example, individuals with untreated OSAS have a three times greater risk of motor vehicle accidents than the general population [3]. OSAS is also associated with an increased risk of cardiovascular disease, diabetes and glucose dysregulation [4], independent from obesity [5].

The first line therapy for OSAS is continuous positive airway pressure (CPAP) [1,6,7]. CPAP has been shown to effectively reduce EDS and to improve daily functioning, cognitive function, mood and quality of life [3,6]. The use of CPAP also reduces traffic accidents [7] and other work-related injuries, and improves work productivity [8]. Although CPAP therapies are highly effective in normalizing AHI and reducing symptoms in symptomatic patients, treatment success is limited by long term nonadherence in nearly half of patients [9]. Technical progress in the systems and interfaces (soundproofing, improved masks, humidification, pressure modulation, etc.) have unfortunately not been sufficient to improve compliance [10,11]. Equally, the effect sizes of telemedicine approaches are not as large as what has been achieved with the use of behavioural therapies, and the impacts on patient and provider satisfaction and cost-effectiveness are not yet clear [12–15].

Nonadherence is related to users' profiles, their representations of OSAS and the benefits they experience from CPAP [12,16,17]. This is why cognitive-behavioural and motivation enhancement therapies conducted by health professionals could be effective in

For peer review only - http://bmjopen.bmj.com/site/about/guidelines.xhtml

ensuring adherence to CPAP. A Cochrane review in 2014 showed that there is a low level of evidence that such interventions increase CPAP use (by 1.44 h per night in six studies; n = 584) and increase the number of participants who used their devices for longer than four hours per night (from 28 to 47% in 3 studies; n= 358)[18]. More robust studies are thus needed to increase the level of evidence regarding these types of interventions.

In addition, patient and public involvement (PPI) is more and more advocated in the fields of health education and research [19–25].Nevertheless, the efficacy of PPI remains to be demonstrated [26]. To our knowledge, only one previous pilot study in 39 patients showed that one-to-one peer support at CPAP initiation was feasible and generated high patient satisfaction. However, the study was not powerful enough to demonstrate effectiveness in terms of adherence to CPAP [18,27]. The data from the study, are, however, useful for designing further studies.

The aim of this adequately powered randomized clinical trial is therefore to assess the role of trained Patient Involvement (PI) representatives to help patients with OSAS to restart CPAP after discontinuation.

METHODS AND ANALYSIS

Study design

This is a prospective, multicentre, randomized controlled trial that will be co-conducted by health professionals, a CPAP home provider and patients as experts or peers or participants. After signing a consent form, patients' participants will be randomized 1.1 to the intervention group with peers or the control group. *Nota bene:* the peers involved in the conduct of the study will sign a confidentiality agreement of non-divulgation of the information exchanged with the participants.

Objectives

Primary research aim

For peer review only

For peer review only - http://bmjopen.bmj.com/site/about/guidelines.xhtml

The primary aim is to evaluate the impact of a 6-month intervention involving trained PI representatives to promote the resumption of CPAP in patients who have discontinued its use. Resumption of CPAP is defined as the medical prescription and the setting up of a new CPAP device at home by the homecare provider.

Secondary research aims:

i) to evaluate the impact of the peer-driven intervention on adherence to CPAP by comparing adherence with the control group (mean adherence and % of nights with at least 4 hours' use /night for 70 % of nights);

ii) to determine the factors associated with the resumption of CPAP treatment;

iii) to assess the satisfaction of the intervention group with the peer-driven intervention at 6 months;

iv) to evaluate the feasibility and the execution of the peer-driven intervention and the satisfaction of peers after the interviews conducted.

Patients, Table 1

Adults with an established diagnosis of severe OSAS (AHI >30 events/hour) who have discontinued CPAP by returning their device to the homecare provider within 4 to 12 months after CPAP initiation will be recruited, Table 1, according to the study flow chart depicted in Figure 1.

Interventions (Figure 1)

Recruitment and training of PI representatives

PI representatives will be recruited from the investigators clinics. To be recruited as a PI representative, patients should:

- have used home CPAP for at least one year,
- have a CPAP adherence of at least 4 h/night for 70% of nights,

For peer review only

- express their motivation in participating in a training and orientation session conducted by research staff and including expert patients from the Grenoble Alpes University Department of Patients (DUP GA) [28],
- accept to conduct 3 motivational sessions by videoconference meetings of 45 to 60 minutes duration with 5 to 8 patients within 6 months after each patient's inclusion,

Patients with any major psychiatric illness, shift-workers or frequent out of town travellers will not be recruited as peers.

Peers will be trained during a three half-days interactive session organised by DUP GA, with experts in patient therapeutic education and communication, and investigators [28]. Peers will be taught how to interact with the patients recruited in the study: the aim is for them to share their experiences but not to provide any medical advice.

Description of the intervention

Trained peers will meet patients randomized into the intervention group by videoconference. Each PI representative will be allocated 5 to 8 patients. They will conduct 3 face to face motivational sessions, each of 45 to 60 minutes duration, over a 6-month period based on the principle of motivational enhancement and cognitive-behavioural therapies [11,13]. The content of the first session is designed to identify and understand the underlying reasons for stopping CPAP treatment and to identify difficulties encountered by the patient (advantages and disadvantages of CPAP treatment). The aim of the second session will be for the patient to define his/her objectives and priorities. During the last session, will be discussed to strengthen the motivation to change and how to plan for it. The peers will receive 100 € per patient for the 3 interviews.

In the control group, patients will be informed, at inclusion, that they can have a visit with a physician investigator at any time to resume treatment if they wish, as is usual practice. At the end of the six-month follow-up period, all patients in both groups will have a consultation with their physician who will suggest they resume CPAP treatment. This visit

For peer review only

may take place earlier if the patient wishes to resume CPAP treatment before the end of the follow-up period. We planned to start inclusions by November 2021 and end the study by December 2023.

Assessment in blinded manner at months 0 and 6

Average adherence to CPAP will be measured from data recorded by the built-in software of the CPAP devices (via tele monitoring or retrieved by a home technician) for 1 month after the final consultation.

The relationship between the variables below and a positive response to the peers intervention (defined by a restart of CPAP treatment) will be analyzed: age, sex, Body Mass Index (BMI), marital status, education level, socio-professional status, precariousness (using the EPICES score), smoking and alcohol use, comorbidities (using Charlson score), history of OSAS (date of diagnosis of OSAS, baseline AHI), observance to treatments (Girerd score), date and reason for stopping CPAP and EDS score (using the Epworth Sleepiness Scale).

To determine patient profiles, their representations of OSAS, their experiences with CPAP and their knowledge and confidence to manage their health, 3 questionnaires will be completed at inclusion (M0) and at the 6-monthfollow-up (M6): the Functional Outcomes of Sleep Questionnaire (FOSQ) a disease-specific quality of life questionnaire [29], the Patient Activation Measure (PAM) a measure that assesses patient knowledge, skill, and confidence for self-management [30] and the Self-Efficacy Measure for Sleep Apnea (SEMSA) [31,32] a tool with strong psychometric properties that identifies patient perceptions that may indicate those most likely not to adhere to treatment.

The satisfaction of participating patients with the PI intervention and the satisfaction of PI representatives will be measured on a 4-point Likert scale: very dissatisfied, dissatisfied, satisfied, very satisfied.

Finally, the feasibility and the execution of the 3 interviews will be assessed by the

For peer review only - http://bmjopen.bmj.com/site/about/guidelines.xhtml

number of interviews carried out in their entirety and the average duration of each interview (in minutes).

All information will be collected in secure electronic medical records in accordance with the requirements of General Data Protection Regulation.

Statistical analysis

Sample size

We hypothesize that 20% of patients allocated to the intervention group will reuse CPAP 6 months as compared to 6% of patients in the control group. A two group χ^2 test with a 5% two-sided significance level will have 80% power to detect such difference between the two groups when the sample size in each group is 90 (nQuery v8, Statistical Solutions, Cork, Ireland). In order to take into account a possible drop-outs and to comply with the intent-to-treat principle, we will inflate the sample size by a factor of 15% [33]. We thus plan to include 104 patients per group (i.e. 208 patients in total). 15 patient peers will be involved.

Feasibility and recruitment

The home care provider, *AGIR* à dom. follows more than 20,000 patients with OSAS who use CPAP in the south of France. In 2018, out of 3,281 patients who started CPAP within the study area (Isère, Savoie and Haute-Savoie), 365 discontinued it between 4 to 12 months post initiation and 6% resumed use within 6 months after discontinuation.

Randomization

After consent, randomization will be performed by a centralized computer software for each investigating center. It will be stratified on the center.

Statistical analysis plan

Descriptive analyses: continuous variables will be expressed as medians (25th/75th percentiles) or means (SD) depending on normality which will be assessed with the Shapiro-Wilk test. Categorical variables will be reported as absolute numbers and

For peer review only - http://bmjopen.bmj.com/site/about/guidelines.xhtml

percentages for both groups. Baseline comparisons between groups will be made using a Student's t-test or Mann-Whitney U test, depending on the distribution. For discrete variables, a $\chi2$ test will be used. If significant differences are observed between arms, ANOVA and multivariable regression will be performed. In the case of missing data, an imputation strategy will be applied according to the percentage of missing values. Data management and statistical analyses will be performed using SAS, V.9.4, SAS Institute.

Primary outcome analysis: the impact of the PI intervention on the resumption of CPAP treatment will be studied by comparing the resumption of CPAP in the 2 arms, using a Chi-square test. To take into account a possible centre effect, a second analysis will be carried out using a conditional logistic regression stratified by the centre; the intervention or control arm will be considered as the dependent variable.

Secondary outcomes analyses: mean CPAP compliance one month after resumption of CPAP will be analysed using a mixed linear model (fixed factor: randomisation arm (intervention vs. control), random factor: centre). Comparison of the probability of resuming CPAP with an average compliance of at least 4 hours/night, 70% of nights between the intervention and control groups will be analysed using a conditional logistic regression, stratified by centre. All analyses will be performed as intention-to-treat and then a sensitivity analysis will also be performed *per protocol* (patients who have not resumed treatment will be considered to have zero adherence).

The association between resumption of CPAP and the sociodemographic parameters, clinical data and the scores of the three questionnaires will be studied by conditional logistic regression models stratified by centre, and adjusted by arm (intervention vs control).

In the intervention arm, descriptive statistics will be presented on the satisfaction as well as on the number of interviews carried out and their average duration.

For peer review only

For peer review only - http://bmjopen.bmj.com/site/about/guidelines.xhtml

Ethics

The study will be conducted in accordance with the Declaration of Helsinki and the recommendations for Good Clinical Practice. Written informed consent will be signed by all study participants before enrolment in the study. Patients will have the right to withdraw from the study without incurring any prejudice at any time.

Patient involvement

RM, first author and expert patient, and members of DUP GA participated in the design of this study and will participate in all stages including teaching peers [28] and promoting and reporting the data, including publication in peer review. Thanks to training with health professionals and expert patients [22,23,25] peers will adopt the appropriate posture to enable patients to find their own resources to overcome barriers to use CPAP.

Dissemination

Dissemination plans of the results include presentations at conferences and a publication in peer-reviewed journal. Updates of the randomized trial will be available at ClinicalTrials.gov. All patients will be informed that the dissemination of results will be accessible on request.

Sponsor and funding

The study sponsor will be AGIR à dom. Co-Principal investigators are RM, an expert patient, and JCB, a researcher. The collaborators and sponsors were not involved in the design of the study and will not influence the execution, analysis or publication of results.

DISCUSSION

OSAS is associated with many negative health consequences [1]. The lack of compliance with home CPAP therapy, which is the first line of treatment, and which has shown to be effective on quality of life is a major issue both in terms of the patient's own health status and in health care utilisation [1,2,7,8]. Attempts have been made to improve CPAP

For peer review only

compliance by improving technical issues relating to the comfort of use of the system [10,11] and the use of the of remote monitoring and telemedicine, along with the implementation of web-based adherence interventions [12–15]; however they have not been shown to improve compliance with the therapy. Other strategies to improve compliance therefore need to be developed and tested.

One of the main strengths of this study is the involvement of peers in the implementation of the behavioural intervention. Regarding efficacy, the involvement of patients with experience in the motivation of their peers to comply with treatment has been implemented with success in other chronic conditions requiring self-management such as HIV and diabetes [34,35]. Furthermore, evidence suggests that patients perceive peers with similar comorbidities as more credible than health-care professionals in the delivery of behavioural interventions [36–38]. The concept of PPI in education and research has been adopted by a growing number of medical schools, particularly in the United kingdom [19,24]. If the results of this study confirm the effectiveness of the PI intervention in promoting resumption of CPAP in patients initially failing CPAP, this study will provide an evidence base to support the use of PI in the management of OSAS in conjunction with the home healthcare provider and specialized sleep centers [39].

The aim to seek factors that are related to CPAP resumption will provide useful information regarding those patients who are more likely to resume CPAP and therefore who PI interventions are more likely to help. This will open the way for further studies to determine the most appropriate methods to improve compliance in those patients who benefit less from PI interventions.

Despite these strengths, the study has two main inherent limitations. Firstly, the results are likely to be biased by the fact that patients who accept to participate may be more likely to resume CPAP therapy than those who decline participation. The results may therefore not be generalizable to all patients who have stopped using their CPAP as prescribed.

For peer review only

For peer review only - http://bmjopen.bmj.com/site/about/guidelines.xhtml

Secondly, the effectiveness of the intervention may also depend on the capacity of the peer-participant to deliver it. The training is quite short (3 half-days) and some of the peers recruited may be more skilled than others in providing such intervention. However, in this study, the peers will be additionally supported throughout the study by the University Department of Patients.

In summary, the results of this study will determine the effectiveness of a PI intervention to motivate patients who have stopped using their CPAP as prescribed to resume its use on compliance with CPAP therapy. The results will also provide information regarding the factors relating to resumption of CPAP, providing a starting point for further studies to determine the most appropriate methods to improve compliance in those patients who benefit less from PI interventions.

For peer review only

Acknowledgements We specially thank John Louis McGregor, PhD, retired Director of Medical Research (DR1) at INSERM, former director of INSERM Unit 331, and retired Honorary Senior Lecturer (research) at the Cardiovascular division King's College London, for his continuous encouragements and counselling together with manuscript reviewing.

Contributors RM participated in the design of the study, wrote the article based on the study protocol, will train PI, collect and analyse data into the protocol. CP participated in the design of the study, wrote the study protocol and will include patients into the protocol together with PPI. SL participated in the design of the study, wrote the study protocol. CD and NA participated in establishing the sample size and will help to recruit patients. MR set up statistical analysis plan and determine sample size. RT revised the manuscript, will include patients into the protocol and collect and analyse data. JLP designed the study, critically revised the manuscript, will include patients, and collect and analyse data. JCB designed the study, critically revised the manuscript and will analyse data. The submitted manuscript has been approved by all authors.

Funding Mr. R. Merle is a recipient of a grant from Agir pour les Maladies Chroniques, http://fonds-apmc.org received in 2019. JLP and RT are supported by the French National Research Agency in the framework of the "Investissements d'avenir" program (ANR-15-IDEX-02) and the "e-health and integrated care and trajectories medicine and MIAI artificial intelligence" Chairs of excellence from the Grenoble-Alpes University Foundation. This work has been partially supported by MIAI @ Grenoble Alpes, (ANR-19-P3IA-0003).

Competing interests Mr. R. Merle is a recipient of a grant from Agir pour les Maladies

For peer review only - http://bmjopen.bmj.com/site/about/guidelines.xhtml

Chroniques, http://fonds-apmc.org/. CD, NA, JCB are employees of *AGIR* à dom. CP and JLP received grants from Agir pour les Maladies Chroniques, http://fonds-apmc.org/.

Ethics approval

The protocol to be approved by The French Regional Ethics Committee CPP Ouest II-Angers.

Provenance and peer review: not commissioned; externally peer reviewed.

Open access This is an open access article distributed in accordance with the Creative Commons Attribution Non Commercial (CC BY-NC 4.0) license, which permits others to distribute, remix, adapt, build upon this work non-commercially, and license their derivative works on different terms, provided the original work is properly cited, appropriate credit is given, any changes made indicated, and the use is non-commercial. See: http:// creativecommons. org/ licenses/ by- nc/ 4.0/.

For peer review only

For peer review only - http://bmjopen.bmj.com/site/about/guidelines.xhtml

REFERENCES

1 Lévy P, Kohler M, McNicholas WT, *et al.* Obstructive sleep apnoea syndrome. *Nat Rev Dis Primer* 2015;**1**:15015. doi:10.1038/nrdp.2015.15

2 Benjafield AV, Ayas NT, Eastwood PR, *et al.* Estimation of the global prevalence and burden of obstructive sleep apnoea: a literature-based analysis. *Lancet Respir Med* 2019;**7**:687–98. doi:10.1016/S2213-2600(19)30198-5

3 Léger D, Stepnowsky C. The economic and societal burden of excessive daytime sleepiness in patients with obstructive sleep apnea. *Sleep Med Rev* 2020;**51**:101275. doi:10.1016/j.smrv.2020.101275

4 Ryan S, Cummins EP, Farre R, *et al.* Understanding the pathophysiological mechanisms of cardiometabolic complications in obstructive sleep apnoea: towards personalised treatment approaches. *Eur Respir J* 2020;**56**. doi:10.1183/13993003.02295-2019

5 Ryan S, Arnaud C, Fitzpatrick SF, *et al.* Adipose tissue as a key player in obstructive sleep apnoea. *Eur Respir Rev Off J Eur Respir Soc* 2019;**28**. doi:10.1183/16000617.0006-2019

6 Wimms AJ, Kelly JL, Turnbull CD, *et al.* Continuous positive airway pressure versus standard care for the treatment of people with mild obstructive sleep apnoea (MERGE): a multicentre, randomised controlled trial. *Lancet Respir Med* 2020;**8**:349–58. doi:10.1016/S2213-2600(19)30402-3

7 George CF. Reduction in motor vehicle collisions following treatment of sleep apnoea with nasal CPAP. *Thorax* 2001;**56**:508–12. doi:10.1136/thorax.56.7.508

For peer review only

For peer review only - http://bmjopen.bmj.com/site/about/guidelines.xhtml

8 Stepnowsky C, Sarmiento KF, Bujanover S, *et al.* Comorbidities, Health-Related Quality of Life, and Work Productivity Among People With Obstructive Sleep Apnea With Excessive Sleepiness: Findings From the 2016 US National Health and Wellness Survey. *J Clin Sleep Med JCSM Off Publ Am Acad Sleep Med* 2019;**15**:235–43. doi:10.5664/jcsm.7624

9 Weaver TE, Grunstein RR. Adherence to continuous positive airway pressure therapy: the challenge to effective treatment. *Proc Am Thorac Soc* 2008;**5**:173–8. doi:10.1513/pats.200708-119MG

10 Donovan LM, Boeder S, Malhotra A, *et al.* New developments in the use of positive airway pressure for obstructive sleep apnea. *J Thorac Dis* 2015;**7**:1323–42. doi:10.3978/j.issn.2072-1439.2015.07.30

11 Rotenberg BW, Murariu D, Pang KP. Trends in CPAP adherence over twenty years of data collection: a flattened curve. *J Otolaryngol - Head Neck Surg* 2016;**45**. doi:10.1186/s40463-016-0156-0

12 Bakker JP, Weaver TE, Parthasarathy S, *et al.* Adherence to CPAP: What Should We Be Aiming For, and How Can We Get There? *Chest* 2019;**155**:1272–87. doi:10.1016/j.chest.2019.01.012

13 Hwang D, Chang JW, Benjafield AV, *et al.* Effect of Telemedicine Education and Telemonitoring on Continuous Positive Airway Pressure Adherence. The Tele-OSA Randomized Trial. *Am J Respir Crit Care Med* 2018;**197**:117–26. doi:10.1164/rccm.201703-0582OC

14 Tamisier R, Treptow E, Joyeux-Faure M, *et al.* Impact of a multimodal telemonitoring intervention on CPAP adherence in symptomatic low-cardiovascular

For peer review only

For peer review only - http://bmjopen.bmj.com/site/about/guidelines.xhtml

risk sleep apnea: a randomized controlled trial. *Chest* Published Online First: 5 July 2020. doi:10.1016/j.chest.2020.05.613

15 Pépin J-L, Jullian-Desayes I, Sapène M, *et al.* Multimodal Remote Monitoring of High Cardiovascular Risk Patients With OSA Initiating CPAP: A Randomized Trial. *Chest* 2019;**155**:730–9. doi:10.1016/j.chest.2018.11.007

16 Weaver TE. Novel Aspects of CPAP Treatment and Interventions to Improve CPAP Adherence. *J Clin Med* 2019;**8**. doi:10.3390/jcm8122220

17 Mendelson M, Gentina T, Gentina E, *et al.* Multidimensional Evaluation of Continuous Positive Airway Pressure (CPAP) Treatment for Sleep Apnea in Different Clusters of Couples. *J Clin Med* 2020;**9**. doi:10.3390/jcm9061658

18 Wozniak DR, Lasserson TJ, Smith I. Educational, supportive and behavioural interventions to improve usage of continuous positive airway pressure machines in adults with obstructive sleep apnoea. *Cochrane Database Syst Rev* Published Online First: 8 January 2014. doi:10.1002/14651858.CD007736.pub2

19 Spencer J, Godolphin W, Karpenko N. *Can patients be teachers? Involving patients and service users in healthcare professionals' education.* https://www.health.org.uk/sites/default/files/CanPatientsBeTeachers.pdf

20 Ocloo J, Matthews R. From tokenism to empowerment: progressing patient and public involvement in healthcare improvement. *BMJ Qual Saf*;**25**:626–32. doi:10.1136/bmjqs-2015-004839

21 Harrison JD, Auerbach AD, Anderson W. Patient stakeholder engagement in research: A narrative review to describe foundational principles and best practice

For peer review only

For peer review only - http://bmjopen.bmj.com/site/about/guidelines.xhtml

activities. *Health Expect Int J Public Particip Health Care Health Policy*;**22**:307–16. doi:10.1111/hex.12873

22 Merle R, Pépin J-L, Ménissier T, *et al.* [Growing role of patients in French health democracy]. *Rev Mal Respir* 2020;**37**:620–3. doi:10.1016/j.rmr.2020.08.002

23 Merle R, Pépin J-L, Palombi O. Successful training of patient and public involvement in health education and clinical research at Grenoble Patients' University centre. *BMJ* 2020.

24 Dijk SW, Duijzer EJ, Wienold M. Role of active patient involvement in undergraduate medical education: a systematic review. *BMJ Open* 2020;**10**:e037217. doi:10.1136/bmjopen-2020-037217

25 Merle R, Casagrande A, Pariset A, *et al.* [The roles of patients in healthcare provision, training and research: A French perspective]. *Rev Epidemiol Sante Publique* Published Online First: 18 May 2021. doi:10.1016/j.respe.2021.04.136

26 Fønhus MS, Dalsbø TK, Johansen M, *et al.* Patient-mediated interventions to improve professional practice. *Cochrane Database Syst Rev* Published Online First: 2018. doi:10.1002/14651858.CD012472.pub2

27 Parthasarathy S, Wendel C, Haynes PL, *et al.* A pilot study of CPAP adherence promotion by peer buddies with sleep apnea. *J Clin Sleep Med JCSM Off Publ Am Acad Sleep Med* 2013;**9**:543–50. doi:10.5664/jcsm.2744

28 Merle R, Pépin J-L, Palombi O, *et al.* Co creation of a programme at Grenoble Patients' University to train patients to teach and participate in research. BMJ Opin. 2021.

For peer review only

29 Weaver TE, Laizner AM, Evans LK, *et al.* An instrument to measure functional status outcomes for disorders of excessive sleepiness. *Sleep* 1997;**20**:835–43.

30 Hibbard JH, Stockard J, Mahoney ER, *et al.* Development of the Patient Activation Measure (PAM): conceptualizing and measuring activation in patients and consumers. *Health Serv Res* 2004;**39**:1005–26. doi:10.1111/j.1475-6773.2004.00269.x

31 Weaver TE, Maislin G, Dinges DF, *et al.* Self-efficacy in sleep apnea: instrument development and patient perceptions of obstructive sleep apnea risk, treatment benefit, and volition to use continuous positive airway pressure. *Sleep* 2003;**26**:727–32. doi:10.1093/sleep/26.6.727

32 Micoulaud-Franchi J-A, Coste O, Bioulac S, *et al.* A French update on the Self-Efficacy Measure for Sleep Apnea (SEMSA) to assess continuous positive airway pressure (CPAP) use. *Sleep Breath Schlaf Atm* 2019;**23**:217–26. doi:10.1007/s11325-018-1686-7

33 Ellenberg SS. Randomization Designs in Comparative Clinical Trials. *N Engl J Med* 1984;**310**:1404–8. doi:10.1056/NEJM198405243102141

34 Deering KN, Shannon K, Sinclair H, *et al.* Piloting a peer-driven intervention model to increase access and adherence to antiretroviral therapy and HIV care among street-entrenched HIV-positive women in Vancouver. *AIDS Patient Care STDs* 2009;**23**:603–9. doi:10.1089/apc.2009.0022

35 Lorig K, Ritter PL, Villa FJ, *et al.* Community-based peer-led diabetes self-management: a randomized trial. *Diabetes Educ* 2009;**35**:641–51. doi:10.1177/0145721709335006

For peer review only

483 36 Heisler M, Halasyamani L, Resnicow K. I Am Not Alone": The Feasibility and Acceptability of Interactive Voice Response-Facilitated Telephone Peer Support Among Older Adults With Heart Failure. *Congest Heart Fail*;**13**:149–57. doi:10.1111/j.1527-5299.2007.06412.x

487 37 Solomon P. Peer support/peer provided services underlying processes, benefits, and critical ingredients. *Psychiatr Rehabil J*;**27**:392–401. doi:10.2975/27.2004.392.401

489 38 Gaines ME, Morris P, Thistlethwaite J. The patient ' s voice in health and social care professional education: The Vancouver Statement. *Int J Health Gov*;**21**:18–25. doi:10.1108/IJHG-01-2016-0003

492 39 Billings ME, Pendharkar SR. Alternative Care Pathways for Obstructive Sleep Apnea and the Impact on Positive Airway Pressure Adherence: Unraveling the Puzzle of Adherence. *Sleep Med Clin* 2020;**0**. doi:10.1016/j.jsmc.2020.10.005

495

496

For peer review only

For peer review only - http://bmjopen.bmj.com/site/about/guidelines.xhtml

Table 1 -Inclusion and exclusion criteria

**Inclusion criteria**	**Exclusion criteria**
▪ Over 18 years' old<br>▪ Diagnosed with of severe OSA (AHI ≥ 30 events/hour)<br>▪ Discontinuation of CPAP 4 to 12 months after initiation, despite the interventions of health professionals and provider, and having stopped their CPAP treatment no later than one year prior to their inclusion<br>▪ Followed by the home health care provider AGIRa*Dom*<br>▪ Access to a computer and/or tablet and an internet connection<br>▪ Oral and written French<br>▪ Able to provide written informed consent<br>▪ Affiliated to social security or beneficiary of such a scheme	▪ CPAP cessation due to a resolution of the OSAS (e.g. weight loss after bariatric surgery) or another pathology that prevents the continuation of treatment (e.g. ENT surgery, etc.)<br>▪ Severe and/or unstable comorbidity that required hospitalization for decompensation in the previous year (heart, kidney, respiratory, liver, psychiatric or other insufficiency)<br>Central sleep apnoea index above 20% of AHI at the time of diagnosis<br>▪ Patient being treated with a mandibular advancement orthosis<br>▪ Lack of availability (e.g. night worker or patient who travels frequently, etc.).<br>▪ Current participation in, participation in the month prior to inclusion in another clinical intervention research study that may impact the study: this impact is left to the investigator's discretion.<br>▪ Referred to in Articles L1121-5 to L1121-8 of the CSP (corresponds to all protected persons: pregnant woman, breastfeeding mother, person deprived of liberty by judicial or administrative decision, person subject to a legal protection measure)

For peer review only

For peer review only - http://bmjopen.bmj.com/site/about/guidelines.xhtml

31-05-2021 version, *BMJ open Protocol-R1, revised document, the 4th of August 2021*

Figure 1. Study design

Supplementary file

SPIRIT 2013 Checklist

Section/item	ItemNo, ligne manuscrit	Description
**Administrative information**		
Title	1, 1-2	Descriptive title identifying the study design, population, interventions, and, if applicable, trial acronym
Trial registration	2a, 62	Trial identifier and registry name. If not yet registered, name of intended registry
	2b,	All items from the World Health Organization Trial Registration Data Set
Protocol version	3, joined, 6-05-2021, v1.1	Date and version identifier
Funding	4, 284-6	Sources and types of financial, material, and other support
Roles and responsibilities	5a, 340-9	Names, affiliations, and roles of protocol contributors
	5b, C. Pison, cpison@chu-grenoble.fr	Name and contact information for the trial sponsor
	5c, none	Role of study sponsor and funders, if any, in study design; collection, management, analysis, and interpretation of data; writing of the report; and the decision to submit the report for publication, including whether they will have ultimate authority over any of these activities
	5d, PI as C. Pison	Composition, roles, and responsibilities of the coordinating centre, steering committee, endpoint adjudication committee, data management team, and other individuals or groups overseeing the trial, if applicable (see Item 21a for data monitoring committee)
**Introduction**		
Background and rationale	6a, 86-124	Description of research question and justification for undertaking the trial, including summary of relevant studies (published and unpublished) examining benefits and harms for each intervention
	6b	Explanation for choice of comparators
Objectives	7, 135-149	Specific objectives or hypotheses

For peer review only

31-05-2021 version, *BMJ open Protocol-R1, revised document, the 4th of August 2021*

Trial design	8, Fig. 1	Description of trial design including type of trial (eg, parallel group, crossover, factorial, single group), allocation ratio, and framework (eg, superiority, equivalence, noninferiority, exploratory)
**Methods: Participants, interventions, and outcomes**		
Study setting	9, 152-5	Description of study settings (eg, community clinic, academic hospital) and list of countries where data will be collected. Reference to where list of study sites can be obtained
Eligibility criteria	10, Table 1	Inclusion and exclusion criteria for participants. If applicable, eligibility criteria for study centres and individuals who will perform the interventions (eg, surgeons, psychotherapists)
Interventions	11a, 157-191	Interventions for each group with sufficient detail to allow replication, including how and when they will be administered
11b, NA		Criteria for discontinuing or modifying allocated interventions for a given trial participant (eg, drug dose change in response to harms, participant request, or improving/worsening disease)
11c, Fig. 1		Strategies to improve adherence to intervention protocols, and any procedures for monitoring adherence (eg, drug tablet return, laboratory tests)
11d, none		Relevant concomitant care and interventions that are permitted or prohibited during the trial
Outcomes	12, 136-149	Primary, secondary, and other outcomes, including the specific measurement variable (eg, systolic blood pressure), analysis metric (eg, change from baseline, final value, time to event), method of aggregation (eg, median, proportion), and time point for each outcome. Explanation of the clinical relevance of chosen efficacy

For peer review only

		and harm outcomes is strongly recommended
Participant timeline	13, Fig. 1	Time schedule of enrolment, interventions (including any run-ins and washouts), assessments, and visits for participants. A schematic diagram is highly recommended (see Figure)
Sample size	14, 220-8	Estimated number of participants needed to achieve study objectives and how it was determined, including clinical and statistical assumptions supporting any sample size calculations
Recruitment	15, 229-33	Strategies for achieving adequate participant enrolment to reach target sample size
**Methods: Assignment of interventions (for controlled trials)**		
Allocation:		
Sequence generation	16a, 235-6	Method of generating the allocation sequence (eg, computer-generated random numbers), and list of any factors for stratification. To reduce predictability of a random sequence, details of any planned restriction (eg, blocking) should be provided in a separate document that is unavailable to those who enrol participants or assign interventions
Allocation concealment mechanism	16b, 235-6	Mechanism of implementing the allocation sequence (eg, central telephone; sequentially numbered, opaque, sealed envelopes), describing any steps to conceal the sequence until interventions are assigned
Implementation	16c, 235-6	Who will generate the allocation sequence, who will enrol participants, and who will assign participants to interventions
Blinding (masking)	17a, NA except outcome assessors	Who will be blinded after assignment to interventions (eg, trial participants, care

For peer review only - http://bmjopen.bmj.com/site/about/guidelines.xhtml

31-05-2021 version, *BMJ open Protocol-R1, revised document, the 4th of August 2021*

		providers, outcome assessors, data analysts), and how
17b, NA		If blinded, circumstances under which unblinding is permissible, and procedure for revealing a participant's allocated intervention during the trial
**Methods: Data collection, management, and analysis**		
Data collection methods	18a	Plans for assessment and collection of outcome, baseline, and other trial data, including any related processes to promote data quality (eg, duplicate measurements, training of assessors) and a description of study instruments (eg, questionnaires, laboratory tests) along with their reliability and validity, if known. Reference to where data collection forms can be found, if not in the protocol
18b, 229-233		Plans to promote participant retention and complete follow-up, including list of any outcome data to be collected for participants who discontinue or deviate from intervention protocols
Data management	19, see Protocol	Plans for data entry, coding, security, and storage, including any related processes to promote data quality (eg, double data entry; range checks for data values). Reference to where details of data management procedures can be found, if not in the protocol
Statistical methods	20a, 237-265	Statistical methods for analysing primary and secondary outcomes. Reference to where other details of the statistical analysis plan can be found, if not in the protocol
20b, NA		Methods for any additional analyses (eg, subgroup and adjusted analyses)
20c, NA		Definition of analysis population relating to protocol non-adherence (eg, as randomised analysis), and any statistical methods to handle missing data (eg, multiple imputation)
**Methods: Monitoring**		

For peer review only

31-05-2021 version, *BMJ open Protocol-R1, revised document, the 4th of August 2021*

Data monitoring	21a, monitoring independant from investigators	Composition of data monitoring committee (DMC); summary of its role and reporting structure; statement of whether it is independent from the sponsor and competing interests; and reference to where further details about its charter can be found, if not in the protocol. Alternatively, an explanation of why a DMC is not needed

For peer review only

31-05-2021 version, *BMJ open Protocol-R1, revised document, the 4th of August 2021*

21b, NA		Description of any interim analyses and stopping guidelines, including who will have access to these interim results and make the final decision to terminate the trial
Harms, NA	22	Plans for collecting, assessing, reporting, and managing solicited and spontaneously reported adverse events and other unintended effects of trial interventions or trial conduct
Auditing	23 every 3 months	Frequency and procedures for auditing trial conduct, if any, and whether the process will be independent from investigators and the sponsor
**Ethics and dissemination**		
Research ethics approval	24, 267-271	Plans for seeking research ethics committee/institutional review board (REC/IRB) approval
Protocol amendments	25, investigators	Plans for communicating important protocol modifications (eg, changes to eligibility criteria, outcomes, analyses) to relevant parties (eg, investigators, REC/IRBs, trial participants, trial registries, journals, regulators)
Consent or assent	26a, patient's doctor	Who will obtain informed consent or assent from potential trial participants or authorised surrogates, and how (see Item 32)
26b, NA		Additional consent provisions for collection and use of participant data and biological specimens in ancillary studies, if applicable
Confidentiality	27,	How personal information about potential and enrolled participants will be collected, shared, and maintained in order to protect confidentiality before, during, and after the trial
Declaration of interests	28, 359-61	Financial and other competing interests for principal investigators for the overall trial and each study site
Access to data	29, investigators	Statement of who will have access to the final trial dataset, and disclosure of

For peer review only

		contractual agreements that limit such access for investigators
Ancillary and post-trial care	30, NA	Provisions, if any, for ancillary and post-trial care, and for compensation to those who suffer harm from trial participation
Dissemination policy	31a, 279-82	Plans for investigators and sponsor to communicate trial results to participants, healthcare professionals, the public, and other relevant groups (eg, via publication, reporting in results databases, or other data sharing arrangements), including any publication restrictions
	31b	Authorship eligibility guidelines and any intended use of professional writers
	31c	Plans, if any, for granting public access to the full protocol, participant-level dataset, and statistical code

**Appendices**		
Informed consent materials	32, protocol	Model consent form and other related documentation given to participants and authorised surrogates
Biological specimens	33, NA	Plans for collection, laboratory evaluation, and storage of biological specimens for genetic or molecular analysis in the current trial and for future use in ancillary studies, if applicable

For peer review only

31-05-2021 version, *BMJ open Protocol-R1, revised document, the 4th of August 2021*

For peer review only

For peer review only - http://bmjopen.bmj.com/site/about/guidelines.xhtml

For peer review only

Figure 1, Work-flow

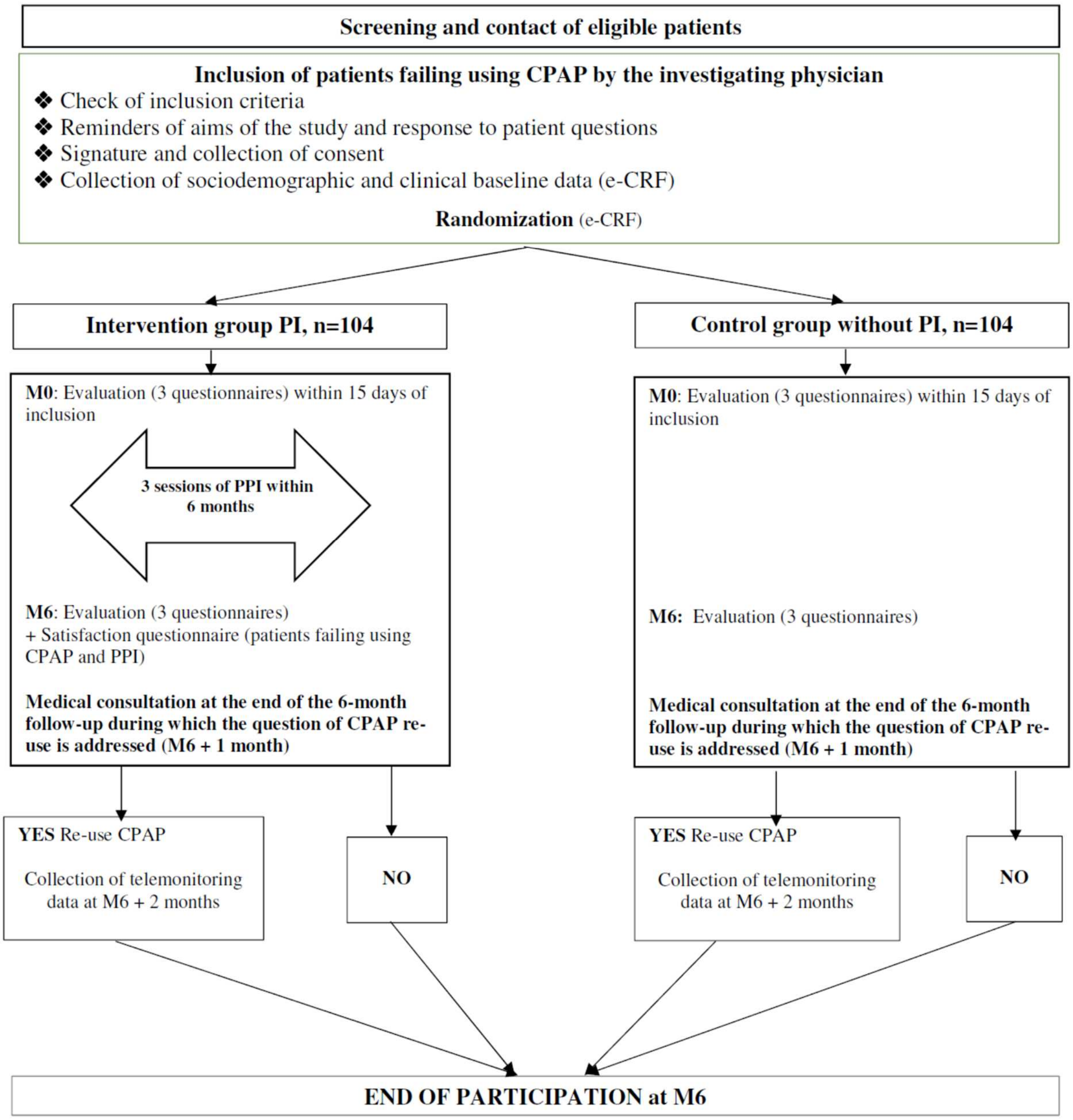

31-05-2021 version, *BMJ open Protocol-R1, revised document, the 4th of August 2021*

SPIRIT 2013 Checklist

**Section/item**	**ItemNo, ligne manuscrit**	**Description**
**Administrative information**		
Title	1, 1-2	Descriptive title identifying the study design, population, interventions, and, if applicable, trial acronym
Trial registration	2a, 62	Trial identifier and registry name. If not yet registered, name of intended registry
2b,		All items from the World Health Organization Trial Registration Data Set
Protocol version	3, joined, 6-05-2021, v1.1	Date and version identifier
Funding	4, 284-6	Sources and types of financial, material, and other support
Roles and responsibilities	5a, 340-9	Names, affiliations, and roles of protocol contributors
5b, C. Pison, cpison@chu-grenoble.fr		Name and contact information for the trial sponsor
5c, none		Role of study sponsor and funders, if any, in study design; collection, management, analysis, and interpretation of data; writing of the report; and the decision to submit the report for publication, including whether they will have ultimate authority over any of these activities
5d, PI as C. Pison		Composition, roles, and responsibilities of the coordinating centre, steering committee, endpoint adjudication committee, data management team, and other individuals or groups overseeing the trial, if applicable (see Item 21a for data monitoring committee)
**Introduction**		
Background and rationale	6a, 86-124	Description of research question and justification for undertaking the trial, including summary of relevant studies (published and unpublished) examining benefits and harms for each intervention
6b		Explanation for choice of comparators
Objectives	7, 135-149	Specific objectives or hypotheses
Trial design	8, Fig. 1	Description of trial design including type of trial (eg, parallel group, crossover, factorial, single group), allocation ratio, and framework (eg, superiority,

For peer review only

			equivalence, noninferiority, exploratory)
**Methods: Participants, interventions, and outcomes**			
Study setting	9, 152-5		Description of study settings (eg, community clinic, academic hospital) and list of countries where data will be collected. Reference to where list of study sites can be obtained
Eligibility criteria	10, Table 1		Inclusion and exclusion criteria for participants. If applicable, eligibility criteria for study centres and individuals who will perform the interventions (eg, surgeons, psychotherapists)
Interventions	11a, 157-191		Interventions for each group with sufficient detail to allow replication, including how and when they will be administered
11b, NA		Criteria for discontinuing or modifying allocated interventions for a given trial participant (eg, drug dose change in response to harms, participant request, or improving/worsening disease)	
11c, Fig. 1		Strategies to improve adherence to intervention protocols, and any procedures for monitoring adherence (eg, drug tablet return, laboratory tests)	
11d, none		Relevant concomitant care and interventions that are permitted or prohibited during the trial	
Outcomes	12, 136-149		Primary, secondary, and other outcomes, including the specific measurement variable (eg, systolic blood pressure), analysis metric (eg, change from baseline, final value, time to event), method of aggregation (eg, median, proportion), and time point for each outcome. Explanation of the clinical relevance of chosen efficacy and harm outcomes is strongly recommended
Participant timeline	13, Fig. 1		Time schedule of enrolment, interventions (including any run-ins and washouts), assessments, and visits for participants. A schematic

For peer review only

31-05-2021 version, *BMJ open Protocol-R1, revised document, the 4th of August 2021*

		diagram is highly recommended (see Figure)
Sample size	14, 220-8	Estimated number of participants needed to achieve study objectives and how it was determined, including clinical and statistical assumptions supporting any sample size calculations
Recruitment	15, 229-33	Strategies for achieving adequate participant enrolment to reach target sample size
**Methods: Assignment of interventions (for controlled trials)**		
Allocation:		
Sequence generation	16a, 235-6	Method of generating the allocation sequence (eg, computer-generated random numbers), and list of any factors for stratification. To reduce predictability of a random sequence, details of any planned restriction (eg, blocking) should be provided in a separate document that is unavailable to those who enrol participants or assign interventions
Allocation concealment mechanism	16b, 235-6	Mechanism of implementing the allocation sequence (eg, central telephone; sequentially numbered, opaque, sealed envelopes), describing any steps to conceal the sequence until interventions are assigned
Implementation	16c, 235-6	Who will generate the allocation sequence, who will enrol participants, and who will assign participants to interventions
Blinding (masking)	17a, NA except outcome assessors	Who will be blinded after assignment to interventions (eg, trial participants, care providers, outcome assessors, data analysts), and how
17b, NA		If blinded, circumstances under which unblinding is permissible, and procedure for revealing a participant's allocated intervention during the trial
**Methods: Data collection, management, and analysis**		

For peer review only

31-05-2021 version, *BMJ open Protocol-R1, revised document, the 4th of August 2021*

Data collection methods	18a	Plans for assessment and collection of outcome, baseline, and other trial data, including any related processes to promote data quality (eg, duplicate measurements, training of assessors) and a description of study instruments (eg, questionnaires, laboratory tests) along with their reliability and validity, if known. Reference to where data collection forms can be found, if not in the protocol
	18b, 229-233	Plans to promote participant retention and complete follow-up, including list of any outcome data to be collected for participants who discontinue or deviate from intervention protocols
Data management	19, see Protocol	Plans for data entry, coding, security, and storage, including any related processes to promote data quality (eg, double data entry; range checks for data values). Reference to where details of data management procedures can be found, if not in the protocol
Statistical methods	20a, 237-265	Statistical methods for analysing primary and secondary outcomes. Reference to where other details of the statistical analysis plan can be found, if not in the protocol
	20b, NA	Methods for any additional analyses (eg, subgroup and adjusted analyses)
	20c, NA	Definition of analysis population relating to protocol non-adherence (eg, as randomised analysis), and any statistical methods to handle missing data (eg, multiple imputation)
**Methods: Monitoring**		
Data monitoring	21a, monitoring independant from investigators	Composition of data monitoring committee (DMC); summary of its role and reporting structure; statement of whether it is independent from the sponsor and competing interests; and reference to where further

For peer review only - http://bmjopen.bmj.com/site/about/guidelines.xhtml

For peer review only

details about its charter can be found, if not in the protocol. Alternatively, an explanation of why a DMC is not needed

3

For peer review only

31-05-2021 version, *BMJ open Protocol-R1, revised document, the 4th of August 2021*

21b, NA		Description of any interim analyses and stopping guidelines, including who will have access to these interim results and make the final decision to terminate the trial
Harms, NA	22	Plans for collecting, assessing, reporting, and managing solicited and spontaneously reported adverse events and other unintended effects of trial interventions or trial conduct
Auditing	23 every 3 months	Frequency and procedures for auditing trial conduct, if any, and whether the process will be independent from investigators and the sponsor
**Ethics and dissemination**		
Research ethics approval	24, 267-271	Plans for seeking research ethics committee/institutional review board (REC/IRB) approval
Protocol amendments	25, investigators	Plans for communicating important protocol modifications (eg, changes to eligibility criteria, outcomes, analyses) to relevant parties (eg, investigators, REC/IRBs, trial participants, trial registries, journals, regulators)
Consent or assent	26a, patient's doctor	Who will obtain informed consent or assent from potential trial participants or authorised surrogates, and how (see Item 32)
26b, NA		Additional consent provisions for collection and use of participant data and biological specimens in ancillary studies, if applicable
Confidentiality	27,	How personal information about potential and enrolled participants will be collected, shared, and maintained in order to protect confidentiality before, during, and after the trial
Declaration of interests	28, 359-61	Financial and other competing interests for principal investigators for the overall trial and each study site
Access to data	29, investigators	Statement of who will have access to the final trial dataset, and disclosure of

For peer review only

		contractual agreements that limit such access for investigators
Ancillary and post-trial care	30, NA	Provisions, if any, for ancillary and post-trial care, and for compensation to those who suffer harm from trial participation
Dissemination policy	31a, 279-82	Plans for investigators and sponsor to communicate trial results to participants, healthcare professionals, the public, and other relevant groups (eg, via publication, reporting in results databases, or other data sharing arrangements), including any publication restrictions
31b		Authorship eligibility guidelines and any intended use of professional writers
31c		Plans, if any, for granting public access to the full protocol, participant-level dataset, and statistical code

**Appendices**		
Informed consent materials	32, protocol	Model consent form and other related documentation given to participants and authorised surrogates
Biological specimens	33, NA	Plans for collection, laboratory evaluation, and storage of biological specimens for genetic or molecular analysis in the current trial and for future use in ancillary studies, if applicable

For peer review only

A peer-driven intervention to help patients resume CPAP therapy following discontinuation: a multicenter, randomized clinical trial with patient involvement

Journal:	*BMJ Open*
Manuscript ID	bmjopen-2021-053996.R2
Article Type:	Protocol
Date Submitted by the Author:	26-Sep-2021

Complete List of Authors:	Merle, Raymond; Université Grenoble Alpes<br>Pison, Christophe; Centre Hospitalier Universitaire Grenoble Alpes, ☐ Service Hospitalier Universitaire Pneumologie Physiologie, Pôle Thorax et Vaisseaux; Université Grenoble Alpes<br>Logerot, Sophie; Agir à dom<br>Deschaux, Chrystèle; AGIR à Dom<br>Arnol, Nathalie; AGIR à Dom<br>Roustit, Matthieu; Centre Hospitalier Universitaire de Grenoble; Universite Grenoble Alpes<br>Tamisier, Renaud; Univ. Grenoble Alpes, HP2; Inserm, U1042; Grenoble Alps University Hospital, EFCR Laboratory<br>Pepin, Jean; Hôpital Universitaire de Grenoble; University Grenoble Alpes<br>BOREL, Jean; Centre Hospitalier Universitaire Grenoble Alpes

**Primary Subject Heading**:	Respiratory medicine
Secondary Subject Heading:	Respiratory medicine
Keywords:	RESPIRATORY MEDICINE (see Thoracic Medicine), SLEEP MEDICINE, Chronic airways disease < THORACIC MEDICINE

I, the Submitting Author has the right to grant and does grant on behalf of all authors of the Work (as defined in the below author licence), an exclusive licence and/or a non-exclusive licence for contributions from authors who are: i) UK Crown employees; ii) where BMJ has agreed a CC-BY licence shall apply, and/or iii) in accordance with the terms applicable for US Federal Government officers or employees acting as part of their official duties; on a worldwide, perpetual, irrevocable, royalty-free basis to BMJ Publishing Group Ltd ("BMJ") its licensees and where the relevant Journal is co-owned by BMJ to the co-owners of the Journal, to publish the Work in this journal and any other BMJ products and to exploit all rights, as set out in our licence.

The Submitting Author accepts and understands that any supply made under these terms is made by BMJ to the Submitting Author unless you are acting as an employee on behalf of your employer or a postgraduate student of an affiliated institution which is paying any applicable article publishing charge ("APC") for Open Access articles. Where the Submitting Author wishes to make the Work available on an Open Access basis (and intends to pay the relevant APC), the terms of reuse of such Open Access shall be governed by a Creative Commons licence – details of these licences and which Creative Commons licence will apply to this Work are set out in our licence referred to above.

Other than as permitted in any relevant BMJ Author's Self Archiving Policies, I confirm this Work has not been accepted for publication elsewhere, is not being considered for publication elsewhere and does not duplicate material already published. I confirm all authors consent to publication of this Work and authorise the granting of this licence.

For peer review only

31-05-2021 version, *BMJ open Protocol-R2, revised document, the18 th of September 2021*

A peer-driven intervention to help patients resume CPAP therapy following discontinuation: a multicenter, randomized clinical trial with patient involvement

Raymond Merle[1,2,3], Christophe Pison[1-4], Sophie Logerot[5,6], Chrystèle Deschaux[5,6], Nathalie Arnol[5,6], Matthieu Roustit[1,7,8], Renaud Tamisier[1,8,9], Jean Louis Pepin[1,4,8*], Jean Christian Borel[5,6,8*]

**co-seniors authors JLP, JCB*

1- Université Grenoble Alpes, France

2- Département Universitaire des Patients Grenoble Alpes, Université Grenoble Alpes, France

3- Laboratoire de Bioénergétique Fondamentale et Appliquée, LBFA, Inserm1055, Saint Martin d'Hères, France

4- Service Hospitalier Universitaire Pneumologie, Pôle Thorax et Vaisseaux, CHU Grenoble Alpes, France

5- *AGIR* à dom. Home assistance and services, Meylan, France

6- *IC*@dom., Investigation Clinique à domicile, Meylan, France

7- CIC Inserm, CHUGA

8- Laboratoire Hypoxie et PhysioPathologies cardiovasculaires et respiratoires, HP2, Inserm1042, Grenoble, France

Corresponding author: Pison, Service Hospitalier Universitaire Pneumologie Physiologie, Pôle Thorax et Vaisseaux, CHU Grenoble Alpes, France, CS10217, 38043 Grenoble Cedex 9, France. Phone + 33 6 83 31 97 81, cpison@chu-grenoble.fr

For peer review only

31-05-2021 version, *BMJ open Protocol-R2, revised document, the18 th of September 2021*

ABSTRACT

Introduction

Obstructive Sleep Apnoea Syndrome (OSAS) is one of the most common chronic diseases. It may be associated with symptoms of excessive daytime sleepiness and neurocognitive and cardiovascular complications. First line therapy for OSAS involves home Continuous Positive Airway Pressure (CPAP), however nearly half of patients do not adhere with this treatment over the long-term. Cognitive-behavioural interventions that include health professionals and patient and public involvement (PPI) are increasingly advocated in the fields of education and research. We hypothesize that a peer-driven intervention could help patients with OSAS to resume CPAP use after discontinuation.

Methods and analysis

We have designed a prospective, multicentre randomized, controlled trial that will be co-conducted by health professionals, a home provider of CPAP and patients as experts or peers or participants. The primary aim is to evaluate the impact of a 6-month, peer-driven intervention to promote the resumption of CPAP after discontinuation. We anticipate that 20% of patients in the intervention group will reuse CPAP as compared to 6% in control group, thus 104 patients must be included in each group. The secondary aims are i) to evaluate the impact of the peer-driven intervention on adherence to CPAP compared to the control group (mean adherence and percentage of nights with at least 4 hours' use /night for 70% of nights); - ii) to determine factors associated with resumption of CPAP; -iii) to assess patient satisfaction with the peer-driven intervention at 6 months; -iv) to evaluate the feasibility and the execution of the peer-driven intervention and peer satisfaction. Adult outpatients with an established diagnosis of severe OSA (Apnea-Hypopnea Index >30 events/hour) that have stopped using CPAP within 4 to 12 months after initiation will be

For peer review only

recruited. The peers who will perform the intervention will be patients with OSAS treated with CPAP with good adherence (at least 4 hours/night, 70% of nights) and trained in motivational enhancement and cognitive-behavioural therapies. Trained peers will conduct 3 interviews within 6 months with participants.

Ethics and dissemination

Ethical approval has been obtained from the French Regional Ethics Committee CPP Ouest II-Angers, (IRB 21.02.25.68606 (2021/25)). All participants will sign written informed consent. The results will be presented at conferences and published in peer-reviewed journals as well as public media.

Trial registration number: NCT04538274

Strengths and limitations of this study

- Patient involvement (PI) from the beginning of the setup of this trial.
- There is a rationale supporting the use of motivational enhancement and cognitive-behavioural therapies performed by peers to promote CPAP resumption.
- Our team has experience in patient and public involvement (PPI) from work undertaken at the Grenoble Alpes University Department of Patients.
- Challenges are to train enough peers with homogenous skills.

Key words: patient and public involvement (PPI), obstructive sleep apnoea syndrome (OSAS), excessive daytime sleepiness, non-adherence, motivational enhancement and cognitive-behavioural therapies

For peer review only - http://bmjopen.bmj.com/site/about/guidelines.xhtml

31-05-2021 version, *BMJ open Protocol-R2, revised document, the18 th of September 2021*

76 **Abbreviations and website addresses**

AGIR à dom.	Home care and services, Meylan, France, https://www.agiradom.com
AHI	Apnoea + Hypopnea Index
CPAP	Continuous Positive Airway Pressure
DUPGA	Département Universitaire des Patients Grenoble Alpes: Grenoble Alpes University Department of Patients, https://medecine.univ-grenoble-alpes.fr/departements/departement-universitaire-des-patients/
EDS	Excessive Daytime Sleepiness
OSAS	Obstructive Sleep Apnoea Syndrome
PI	Patient Involvement
PPI	Patient and Public Involvement

77

78

For peer review only - http://bmjopen.bmj.com/site/about/guidelines.xhtml

31-05-2021 version, *BMJ open Protocol-R2, revised document, the18 th of September 2021*

INTRODUCTION

Obstructive Sleep Apnoea syndrome (OSAS) is one of the most common chronic diseases. It is characterized by recurrent episodes of upper airway collapse during sleep, and may or may not be associated symptoms of excessive daytime sleepiness (EDS) and neurocognitive and cardiovascular complications [1]. Twelve million adults aged between 30 and 69 years may have moderate to severe OSAS in France, based on an Apnoea Hypopnea Index (AHI) threshold value of 15 or more events per hour of sleep [2]. The risks associated with the disease can be severe, for example, individuals with untreated OSAS have a three times greater risk of motor vehicle accidents than the general population [3]. OSAS is also associated with an increased risk of cardiovascular disease, diabetes and glucose dysregulation [4], independent from obesity [5].

The first line therapy for OSAS is continuous positive airway pressure (CPAP) [1,6,7]. CPAP has been shown to effectively reduce EDS and to improve daily functioning, cognitive function, mood and quality of life [3,6]. The use of CPAP also reduces traffic accidents [7] and other work-related injuries, and improves work productivity [8]. Although CPAP therapies are highly effective in normalizing AHI and reducing symptoms in symptomatic patients, treatment success is limited by long term nonadherence in nearly half of patients [9]. Technical progress in the systems and interfaces (soundproofing, improved masks, humidification, pressure modulation, etc.) have unfortunately not been sufficient to improve compliance [10,11]. Equally, the effect sizes of telemedicine approaches are not as large as what has been achieved with the use of behavioural therapies, and the impacts on patient and provider satisfaction and cost-effectiveness are not yet clear [12–15].

Nonadherence is related to users' profiles, their representations of OSAS and the benefits they experience from CPAP [12,16,17]. This is why cognitive-behavioural and

For peer review only - http://bmjopen.bmj.com/site/about/guidelines.xhtml

motivation enhancement therapies conducted by health professionals could be effective in ensuring adherence to CPAP. A Cochrane review in 2014 showed that there is a low level of evidence that such interventions increase CPAP use (by 1.44 h per night in six studies; n = 584) and increase the number of participants who used their devices for longer than four hours per night (from 28 to 47% in 3 studies; n= 358)[18]. More robust studies are thus needed to increase the level of evidence regarding these types of interventions.

In addition, patient and public involvement (PPI) is more and more advocated in the fields of health education and research [19–25]. Nevertheless, the efficacy of PPI remains to be demonstrated [26]. To our knowledge, only one previous pilot study in 39 patients showed that one-to-one peer support at CPAP initiation was feasible and generated high patient satisfaction. However, the study was not powerful enough to demonstrate effectiveness in terms of adherence to CPAP [18,27]. The data from the study, are, however, useful for designing further studies.

The aim of this adequately powered randomized clinical trial is therefore to assess the role of trained Patient Involvement (PI) representatives to help patients with OSAS to restart CPAP after discontinuation.

METHODS AND ANALYSIS

Study design

This is a prospective, multicentre, randomized controlled trial that will be co-conducted by health professionals, a CPAP home provider and patients as experts or peers or participants. After signing a consent form, patients' participants will be randomized 1.1 to the intervention group with peers or the control group. *Nota bene:* the peers involved in the conduct of the study will sign a confidentiality agreement of non-divulgation of the information exchanged with the participants.

For peer review only

Objectives

Primary research aim

The primary aim is to evaluate the impact of a 6-month intervention involving trained PI representatives to promote the resumption of CPAP in patients who have discontinued its use.

Primary research outcome

Resumption of CPAP is defined as the medical prescription and the setting up of a new CPAP device at home by the homecare provider.

Secondary research aims:

i) to evaluate the impact of the peer-driven intervention on adherence to CPAP by comparing adherence with the control group (mean adherence and % of nights with at least 4 hours' use /night for 70 % of nights);

ii) to determine the factors associated with the resumption of CPAP treatment;

iii) to assess the satisfaction of the intervention group with the peer-driven intervention at 6 months;

iv) to evaluate the feasibility and the execution of the peer-driven intervention and the satisfaction of peers after the interviews conducted.

Secondary research outcomes

i. average adherence to CPAP will be measured from data recorded by the built-in software of the CPAP devices (via tele monitoring or retrieved by a home technician) for 1 month after the final consultation.

ii. the relationship between the variables below and a positive response to the peers intervention (defined by a restart of CPAP treatment) will be analyzed: age, gender, Body Mass Index (BMI), marital status and number of young children (<10 years) education level, socio-professional status, fragility and social precariousness (using the

For peer review only - http://bmjopen.bmj.com/site/about/guidelines.xhtml

EPICES score), smoking and alcohol use, comorbidities (using Charlson score), history of OSAS (date of diagnosis of OSAS, baseline AHI), observance to treatments (Girerd score), date and reason for stopping CPAP and EDS score (using the Epworth Sleepiness Scale). To determine patient profiles, their representations of OSAS, their experiences with CPAP and their knowledge and confidence to manage their health, 3 questionnaires will be completed at inclusion (M0) and at the 6-monthfollow-up (M6): the Functional Outcomes of Sleep Questionnaire (FOSQ) a disease-specific quality of life questionnaire [28], the Patient Activation Measure (PAM) a measure that assesses patient knowledge, skill, and confidence for self-management [29] and the Self-Efficacy Measure for Sleep Apnea (SEMSA) [30,31] a tool with strong psychometric properties that identifies patient perceptions that may indicate those most likely not to adhere to treatment.

iii. the satisfaction of participating patients with the PI intervention and the satisfaction of PI representatives will be measured on a 4-point Likert scale: very dissatisfied, dissatisfied, satisfied, very satisfied.

iv. the feasibility and the execution of the 3 interviews will be assessed by the number of interviews carried out in their entirety and the average duration of each interview (in minutes).

All information will be collected in secure electronic medical records in accordance with the requirements of General Data Protection Regulation.

Patients, Table 1

Adults with an established diagnosis of severe OSAS (AHI >30 events/hour) who have discontinued CPAP by returning their device to the homecare provider within 4 to 12 months after CPAP initiation will be recruited according to the study flow chart depicted

For peer review only - http://bmjopen.bmj.com/site/about/guidelines.xhtml

in Figure 1.

Interventions (Figure 1)

Recruitment and training of PI representatives

PI representatives will be recruited from the investigators clinics. To be recruited as a PI representative, patients should, Table I:

- have used home CPAP for at least one year,
- have a CPAP adherence of at least 4 h/night for 70% of nights,
- express their motivation in participating in a training and orientation session conducted by research staff and including expert patients from the Grenoble Alpes University Department of Patients (DUP GA) [23],
- accept to conduct 3 motivational sessions by videoconference meetings of 45 to 60 minutes duration with 5 to 8 patients within 6 months after each patient's inclusion,

Patients with any major psychiatric illness, shift-workers or frequent out of town travellers will not be recruited as peers.

Peers will be trained during a three half-days interactive session organised by DUP GA, with experts in patient therapeutic education and communication, and investigators [23]. Peers will be taught how to interact with the patients recruited in the study: the aim is for them to share their experiences but not to provide any medical advice.

Description of the intervention

Trained peers will meet patients randomized into the intervention group by videoconference. Each PI representative will be allocated 5 to 8 patients. They will conduct 3 face to face motivational sessions, each of 45 to 60 minutes duration, over a 6-month period based on the principle of motivational enhancement and cognitive-behavioural therapies [11,13]. The content of the first session is designed to identify and understand the

For peer review only - http://bmjopen.bmj.com/site/about/guidelines.xhtml

underlying reasons for stopping CPAP treatment and to identify difficulties encountered by the patient (advantages and disadvantages of CPAP treatment). The aim of the second session will be for the patient to define his/her objectives and priorities. During the last session, will be discussed to strengthen the motivation to change and how to plan for it. The peers will receive 100 € per patient for the 3 interviews.

In the control group, patients will be informed, at inclusion, that they can have a visit with a physician investigator at any time to resume treatment if they wish, as is usual practice. At the end of the six-month follow-up period, all patients in both groups will have a consultation with their physician who will suggest they resume CPAP treatment. This visit may take place earlier if the patient wishes to resume CPAP treatment before the end of the follow-up period. We planned to start inclusions by November 2021 and end the study by December 2023.

Statistical analysis

Sample size

We hypothesize that 20% of patients allocated to the intervention group will reuse CPAP 6 months as compared to 6% of patients in the control group. A two group χ^2 test with a 5% two-sided significance level will have 80% power to detect such difference between the two groups when the sample size in each group is 90 (nQuery v8, Statistical Solutions, Cork, Ireland). In order to take into account a possible drop-outs and to comply with the intent-to-treat principle, we will inflate the sample size by a factor of 15% [32]. We thus plan to include 104 patients per group (i.e. 208 patients in total). 15 patient peers will be involved.

Feasibility and recruitment

The home care provider, *AGIR* à dom. follows more than 20,000 patients with OSAS who use CPAP in the south of France. In 2018, out of 3,281 patients who started CPAP

For peer review only - http://bmjopen.bmj.com/site/about/guidelines.xhtml

within the study area (Isère, Savoie and Haute-Savoie), 365 discontinued it between 4 to 12 months post initiation and 6% resumed use within 6 months after discontinuation.

Randomization

After consent, randomization will be performed by a centralized computer software for each investigating center. It will be stratified on the center.

Statistical analysis plan

Descriptive analyses: continuous variables will be expressed as medians (25th/75th percentiles) or means (SD) depending on normality which will be assessed with the Shapiro-Wilk test. Categorical variables will be reported as absolute numbers and percentages for both groups. Baseline comparisons between groups will be made using a Student's t-test or Mann-Whitney U test, depending on the distribution. For discrete variables, a $\chi 2$ test will be used. If significant differences are observed between arms, ANOVA and multivariable regression will be performed. In the case of missing data, an imputation strategy will be applied according to the percentage of missing values. Data management and statistical analyses will be performed using SAS, V.9.4, SAS Institute.

Primary outcome analysis: the impact of the PI intervention on the resumption of CPAP treatment will be studied by comparing the resumption of CPAP in the 2 arms, using a Chi-square test. To take into account a possible centre effect, a second analysis will be carried out using a conditional logistic regression stratified by the centre; the intervention or control arm will be considered as the dependent variable.

Secondary outcomes analyses: mean CPAP compliance one month after resumption of CPAP will be analysed using a mixed linear model (fixed factor: randomisation arm (intervention vs. control), random factor: centre). Comparison of the probability of resuming CPAP with an average compliance of at least 4 hours/night, 70% of nights between the intervention and control groups will be analysed using a conditional logistic

For peer review only - http://bmjopen.bmj.com/site/about/guidelines.xhtml

regression, stratified by centre. All analyses will be performed as intention-to-treat and then a sensitivity analysis will also be performed *per protocol* (patients who have not resumed treatment will be considered to have zero adherence).

The association between resumption of CPAP and the sociodemographic parameters, clinical data and the scores of the three questionnaires will be studied by conditional logistic regression models stratified by centre, and adjusted by arm (intervention vs control).

In the intervention arm, descriptive statistics will be presented on the satisfaction as well as on the number of interviews carried out and their average duration.

Ethics

The study will be conducted in accordance with the Declaration of Helsinki and the recommendations for Good Clinical Practice. Written informed consent will be signed by all study participants before enrolment in the study. Patients will have the right to withdraw from the study without incurring any prejudice at any time.

Patient involvement

RM, first author and expert patient, and members of DUP GA participated in the design of this study and will participate in all stages including teaching peers [23] and promoting and reporting the data, including publication in peer review. Thanks to training with health professionals and expert patients [22,23,25] peers will adopt the appropriate posture to enable patients to find their own resources to overcome barriers to use CPAP.

Dissemination

Dissemination plans of the results include presentations at conferences and a publication in peer-reviewed journal. Updates of the randomized trial will be available at ClinicalTrials.gov. All patients will be informed that the dissemination of results will be

For peer review only

accessible on request.

Sponsor and funding

The study sponsor will be AGIR à dom. Co-Principal investigators are RM, an expert patient, and JCB, a researcher. The collaborators and sponsors were not involved in the design of the study and will not influence the execution, analysis or publication of results.

DISCUSSION

OSAS is associated with many negative health consequences [1]. The lack of compliance with home CPAP therapy, which is the first line of treatment, and which has shown to be effective on quality of life is a major issue both in terms of the patient's own health status and in health care utilisation [1,2,7,8]. Attempts have been made to improve CPAP compliance by improving technical issues relating to the comfort of use of the system [10,11] and the use of the of remote monitoring and telemedicine, along with the implementation of web-based adherence interventions [12–15]; however they have not been shown to improve compliance with the therapy. Other strategies to improve compliance therefore need to be developed and tested.

One of the main strengths of this study is the involvement of peers in the implementation of the behavioural intervention. Regarding efficacy, the involvement of patients with experience in the motivation of their peers to comply with treatment has been implemented with success in other chronic conditions requiring self-management such as HIV and diabetes [33,34]. Furthermore, evidence suggests that patients perceive peers with similar comorbidities as more credible than health-care professionals in the delivery of behavioural interventions [35–37]. The concept of PPI in education and research has been adopted by a growing number of medical schools, particularly in the United kingdom [19,24]. If the results of this study confirm the effectiveness of the PI intervention in promoting

For peer review only - http://bmjopen.bmj.com/site/about/guidelines.xhtml

resumption of CPAP in patients initially failing CPAP, this study will provide an evidence base to support the use of PI in the management of OSAS in conjunction with the home healthcare provider and specialized sleep centers [38].

The aim to seek factors that are related to CPAP resumption will provide useful information regarding those patients who are more likely to resume CPAP and therefore who PI interventions are more likely to help. This will open the way for further studies to determine the most appropriate methods to improve compliance in those patients who benefit less from PI interventions.

Despite these strengths, the study has two main inherent limitations. Firstly, the results are likely to be biased by the fact that patients who accept to participate may be more likely to resume CPAP therapy than those who decline participation. The results may therefore not be generalizable to all patients who have stopped using their CPAP as prescribed. Secondly, the effectiveness of the intervention may also depend on the capacity of the peer-participant to deliver it. The training is quite short (3 half-days) and some of the peers recruited may be more skilled than others in providing such intervention. However, in this study, the peers will be additionally supported throughout the study by the University Department of Patients.

In summary, the results of this study will determine the effectiveness of a PI intervention to motivate patients who have stopped using their CPAP as prescribed to resume its use on compliance with CPAP therapy. The results will also provide information regarding the factors relating to resumption of CPAP, providing a starting point for further studies to determine the most appropriate methods to improve compliance in those patients who benefit less from PI interventions.

For peer review only

For peer review only

For peer review only - http://bmjopen.bmj.com/site/about/guidelines.xhtml

31-05-2021 version, *BMJ open Protocol-R2, revised document, the18 th of September 2021*

Acknowledgements We specially thank John Louis McGregor, PhD, retired Director of Medical Research (DR1) at INSERM, former director of INSERM Unit 331, and retired Honorary Senior Lecturer (research) at the Cardiovascular division King's College London, for his continuous encouragements and counselling together with manuscript reviewing.

Contributors RM participated in the design of the study, wrote the article based on the study protocol, will train PI, collect and analyse data into the protocol. CP participated in the design of the study, wrote the study protocol and will include patients into the protocol together with PPI. SL participated in the design of the study, wrote the study protocol. CD and NA participated in establishing the sample size and will help to recruit patients. MR set up statistical analysis plan and determine sample size. RT revised the manuscript, will include patients into the protocol and collect and analyse data. JLP designed the study, critically revised the manuscript, will include patients, and collect and analyse data. JCB designed the study, critically revised the manuscript and will analyse data. The submitted manuscript has been approved by all authors.

Funding Mr. R. Merle is a recipient of a grant from Agir pour les Maladies Chroniques, http://fonds-apmc.org received in 2019. JLP and RT are supported by the French National Research Agency in the framework of the "Investissements d'avenir" program (ANR-15-IDEX-02) and the "e-health and integrated care and trajectories medicine and MIAI artificial intelligence" Chairs of excellence from the Grenoble-Alpes University Foundation. This work has been partially supported by MIAI @ Grenoble Alpes, (ANR-19-P3IA-0003).

For peer review only

For peer review only - http://bmjopen.bmj.com/site/about/guidelines.xhtml

Competing interests Mr. R. Merle is a recipient of a grant from Agir pour les Maladies Chroniques, http://fonds-apmc.org/. CD, NA, JCB are employees of *AGIR* à dom. CP and JLP received grants from Agir pour les Maladies Chroniques, http://fonds-apmc.org/.

Ethics approval

The protocol to be approved by The French Regional Ethics Committee CPP Ouest II-Angers.

Provenance and peer review: not commissioned; externally peer reviewed.

Open access This is an open access article distributed in accordance with the Creative Commons Attribution Non Commercial (CC BY-NC 4.0) license, which permits others to distribute, remix, adapt, build upon this work non-commercially, and license their derivative works on different terms, provided the original work is properly cited, appropriate credit is given, any changes made indicated, and the use is non-commercial. See: http:// creativecommons. org/ licenses/ by- nc/ 4.0/.

For peer review only

31-05-2021 version, *BMJ open Protocol-R2, revised document, the18 th of September 2021*

REFERENCES

1 Lévy P, Kohler M, McNicholas WT, *et al.* Obstructive sleep apnoea syndrome. *Nat Rev Dis Primer* 2015;**1**:15015. doi:10.1038/nrdp.2015.15

2 Benjafield AV, Ayas NT, Eastwood PR, *et al.* Estimation of the global prevalence and burden of obstructive sleep apnoea: a literature-based analysis. *Lancet Respir Med* 2019;**7**:687–98. doi:10.1016/S2213-2600(19)30198-5

3 Léger D, Stepnowsky C. The economic and societal burden of excessive daytime sleepiness in patients with obstructive sleep apnea. *Sleep Med Rev* 2020;**51**:101275. doi:10.1016/j.smrv.2020.101275

4 Ryan S, Cummins EP, Farre R, *et al.* Understanding the pathophysiological mechanisms of cardiometabolic complications in obstructive sleep apnoea: towards personalised treatment approaches. *Eur Respir J* 2020;**56**. doi:10.1183/13993003.02295-2019

5 Ryan S, Arnaud C, Fitzpatrick SF, *et al.* Adipose tissue as a key player in obstructive sleep apnoea. *Eur Respir Rev Off J Eur Respir Soc* 2019;**28**. doi:10.1183/16000617.0006-2019

6 Wimms AJ, Kelly JL, Turnbull CD, *et al.* Continuous positive airway pressure versus standard care for the treatment of people with mild obstructive sleep apnoea (MERGE): a multicentre, randomised controlled trial. *Lancet Respir Med* 2020;**8**:349–58. doi:10.1016/S2213-2600(19)30402-3

7 George CF. Reduction in motor vehicle collisions following treatment of sleep apnoea with nasal CPAP. *Thorax* 2001;**56**:508–12. doi:10.1136/thorax.56.7.508

8 Stepnowsky C, Sarmiento KF, Bujanover S, *et al.* Comorbidities, Health-Related Quality of Life, and Work Productivity Among People With Obstructive Sleep Apnea With Excessive Sleepiness: Findings From the 2016 US National Health and Wellness Survey. *J Clin Sleep Med JCSM Off Publ Am Acad Sleep Med* 2019;**15**:235–43. doi:10.5664/jcsm.7624

9 Weaver TE, Grunstein RR. Adherence to continuous positive airway pressure therapy: the challenge to effective treatment. *Proc Am Thorac Soc* 2008;**5**:173–8. doi:10.1513/pats.200708-119MG

10 Donovan LM, Boeder S, Malhotra A, *et al.* New developments in the use of positive airway pressure for obstructive sleep apnea. *J Thorac Dis* 2015;**7**:1323–42. doi:10.3978/j.issn.2072-1439.2015.07.30

11 Rotenberg BW, Murariu D, Pang KP. Trends in CPAP adherence over twenty years of data collection: a flattened curve. *J Otolaryngol - Head Neck Surg* 2016;**45**. doi:10.1186/s40463-016-0156-0

12 Bakker JP, Weaver TE, Parthasarathy S, *et al.* Adherence to CPAP: What Should We Be Aiming For, and How Can We Get There? *Chest* 2019;**155**:1272–87. doi:10.1016/j.chest.2019.01.012

For peer review only - http://bmjopen.bmj.com/site/about/guidelines.xhtml

13 Hwang D, Chang JW, Benjafield AV, *et al.* Effect of Telemedicine Education and Telemonitoring on Continuous Positive Airway Pressure Adherence. The Tele-OSA Randomized Trial. *Am J Respir Crit Care Med* 2018;**197**:117–26. doi:10.1164/rccm.201703-0582OC

14 Tamisier R, Treptow E, Joyeux-Faure M, *et al.* Impact of a multimodal telemonitoring intervention on CPAP adherence in symptomatic low-cardiovascular risk sleep apnea: a randomized controlled trial. *Chest* Published Online First: 5 July 2020. doi:10.1016/j.chest.2020.05.613

15 Pépin J-L, Jullian-Desayes I, Sapène M, *et al.* Multimodal Remote Monitoring of High Cardiovascular Risk Patients With OSA Initiating CPAP: A Randomized Trial. *Chest* 2019;**155**:730–9. doi:10.1016/j.chest.2018.11.007

16 Weaver TE. Novel Aspects of CPAP Treatment and Interventions to Improve CPAP Adherence. *J Clin Med* 2019;**8**. doi:10.3390/jcm8122220

17 Mendelson M, Gentina T, Gentina E, *et al.* Multidimensional Evaluation of Continuous Positive Airway Pressure (CPAP) Treatment for Sleep Apnea in Different Clusters of Couples. *J Clin Med* 2020;**9**. doi:10.3390/jcm9061658

18 Wozniak DR, Lasserson TJ, Smith I. Educational, supportive and behavioural interventions to improve usage of continuous positive airway pressure machines in adults with obstructive sleep apnoea. *Cochrane Database Syst Rev* Published Online First: 8 January 2014. doi:10.1002/14651858.CD007736.pub2

19 Spencer J, Godolphin W, Karpenko N. *Can patients be teachers? Involving patients and service users in healthcare professionals' education.* https://www.health.org.uk/sites/default/files/CanPatientsBeTeachers.pdf

20 Ocloo J, Matthews R. From tokenism to empowerment: progressing patient and public involvement in healthcare improvement. *BMJ Qual Saf*;**25**:626–32. doi:10.1136/bmjqs-2015-004839

21 Harrison JD, Auerbach AD, Anderson W. Patient stakeholder engagement in research: A narrative review to describe foundational principles and best practice activities. *Health Expect Int J Public Particip Health Care Health Policy*;**22**:307–16. doi:10.1111/hex.12873

22 Merle R, Pépin J-L, Ménissier T, *et al.* [Growing role of patients in French health democracy]. *Rev Mal Respir* 2020;**37**:620–3. doi:10.1016/j.rmr.2020.08.002

23 Merle R. A co-led training programme to support patients to take on key roles in teaching and advancing active patient and public involvement. The BMJ. 2021.https://blogs.bmj.com/bmj/2021/02/22/a-co-led-training-programme-to-support-patients-to-take-on-key-roles-in-teaching-and-advancing-active-patient-and-public-involvement/ (accessed 18 Sep 2021).

24 Dijk SW, Duijzer EJ, Wienold M. Role of active patient involvement in undergraduate medical education: a systematic review. *BMJ Open* 2020;**10**:e037217. doi:10.1136/bmjopen-2020-037217

For peer review only - http://bmjopen.bmj.com/site/about/guidelines.xhtml

For peer review only

25 Merle R, Casagrande A, Pariset A, *et al.* [The roles of patients in healthcare provision, training and research: A French perspective]. *Rev Epidemiol Sante Publique* Published Online First: 18 May 2021. doi:10.1016/j.respe.2021.04.136

26 Fønhus MS, Dalsbø TK, Johansen M, *et al.* Patient-mediated interventions to improve professional practice. *Cochrane Database Syst Rev* Published Online First: 2018. doi:10.1002/14651858.CD012472.pub2

27 Parthasarathy S, Wendel C, Haynes PL, *et al.* A pilot study of CPAP adherence promotion by peer buddies with sleep apnea. *J Clin Sleep Med JCSM Off Publ Am Acad Sleep Med* 2013;**9**:543–50. doi:10.5664/jcsm.2744

28 Weaver TE, Laizner AM, Evans LK, *et al.* An instrument to measure functional status outcomes for disorders of excessive sleepiness. *Sleep* 1997;**20**:835–43.

29 Hibbard JH, Stockard J, Mahoney ER, *et al.* Development of the Patient Activation Measure (PAM): conceptualizing and measuring activation in patients and consumers. *Health Serv Res* 2004;**39**:1005–26. doi:10.1111/j.1475-6773.2004.00269.x

30 Weaver TE, Maislin G, Dinges DF, *et al.* Self-efficacy in sleep apnea: instrument development and patient perceptions of obstructive sleep apnea risk, treatment benefit, and volition to use continuous positive airway pressure. *Sleep* 2003;**26**:727–32. doi:10.1093/sleep/26.6.727

31 Micoulaud-Franchi J-A, Coste O, Bioulac S, *et al.* A French update on the Self-Efficacy Measure for Sleep Apnea (SEMSA) to assess continuous positive airway pressure (CPAP) use. *Sleep Breath Schlaf Atm* 2019;**23**:217–26. doi:10.1007/s11325-018-1686-7

32 Ellenberg SS. Randomization Designs in Comparative Clinical Trials. *N Engl J Med* 1984;**310**:1404–8. doi:10.1056/NEJM198405243102141

33 Deering KN, Shannon K, Sinclair H, *et al.* Piloting a peer-driven intervention model to increase access and adherence to antiretroviral therapy and HIV care among street-entrenched HIV-positive women in Vancouver. *AIDS Patient Care STDs* 2009;**23**:603–9. doi:10.1089/apc.2009.0022

34 Lorig K, Ritter PL, Villa FJ, *et al.* Community-based peer-led diabetes self-management: a randomized trial. *Diabetes Educ* 2009;**35**:641–51. doi:10.1177/0145721709335006

35 Heisler M, Halasyamani L, Resnicow K. I Am Not Alone": The Feasibility and Acceptability of Interactive Voice Response-Facilitated Telephone Peer Support Among Older Adults With Heart Failure. *Congest Heart Fail*;**13**:149–57. doi:10.1111/j.1527-5299.2007.06412.x

36 Solomon P. Peer support/peer provided services underlying processes, benefits, and critical ingredients. *Psychiatr Rehabil J*;**27**:392–401. doi:10.2975/27.2004.392.401

For peer review only

37 Gaines ME, Morris P, Thistlethwaite J. The patient ’ s voice in health and social care professional education: The Vancouver Statement. *Int J Health Gov*;**21**:18–25. doi:10.1108/IJHG-01-2016-0003

38 Billings ME, Pendharkar SR. Alternative Care Pathways for Obstructive Sleep Apnea and the Impact on Positive Airway Pressure Adherence: Unraveling the Puzzle of Adherence. *Sleep Med Clin* 2020;**0**. doi:10.1016/j.jsmc.2020.10.005

For peer review only

31-05-2021 version, *BMJ open Protocol-R2, revised document, the18 th of September 2021*

Table 1 -Inclusion and exclusion criteria

**Inclusion criteria**	**Exclusion criteria**
▪ Over 18 years' old<br>▪ Diagnosed with of severe OSA (AHI ≥ 30 events/hour)<br>▪ Discontinuation of CPAP 4 to 12 months after initiation, despite the interventions of health professionals and provider, and having stopped their CPAP treatment no later than one year prior to their inclusion<br>▪ Followed by the home health care provider AGIRa*Dom*<br>▪ Access to a computer and/or tablet and an internet connection<br>▪ Oral and written French<br>▪ Able to provide written informed consent<br>▪ Affiliated to social security or beneficiary of such a scheme	▪ CPAP cessation due to a resolution of the OSAS (e.g. weight loss after bariatric surgery) or another pathology that prevents the continuation of treatment (e.g. ENT surgery, etc.)<br>▪ Severe and/or unstable comorbidity that required hospitalization for decompensation in the previous year (heart, kidney, respiratory, liver, psychiatric or other insufficiency)<br>Central sleep apnoea index above 20% of AHI at the time of diagnosis<br>▪ Patient being treated with a mandibular advancement orthosis<br>▪ Lack of availability (e.g. night worker or patient who travels frequently, etc.).<br>▪ Current participation in, participation in the month prior to inclusion in another clinical intervention research study that may impact the study: this impact is left to the investigator's discretion.<br>▪ Referred to in Articles L1121-5 to L1121-8 of the CSP (corresponds to all protected persons: pregnant woman, breastfeeding mother, person deprived of liberty by judicial or administrative decision, person subject to a legal protection measure)

For peer review only

31-05-2021 version, *BMJ open Protocol-R2, revised document, the18 th of September 2021*

Figure 1. Study design

For peer review only

Figure 1, Work-flow

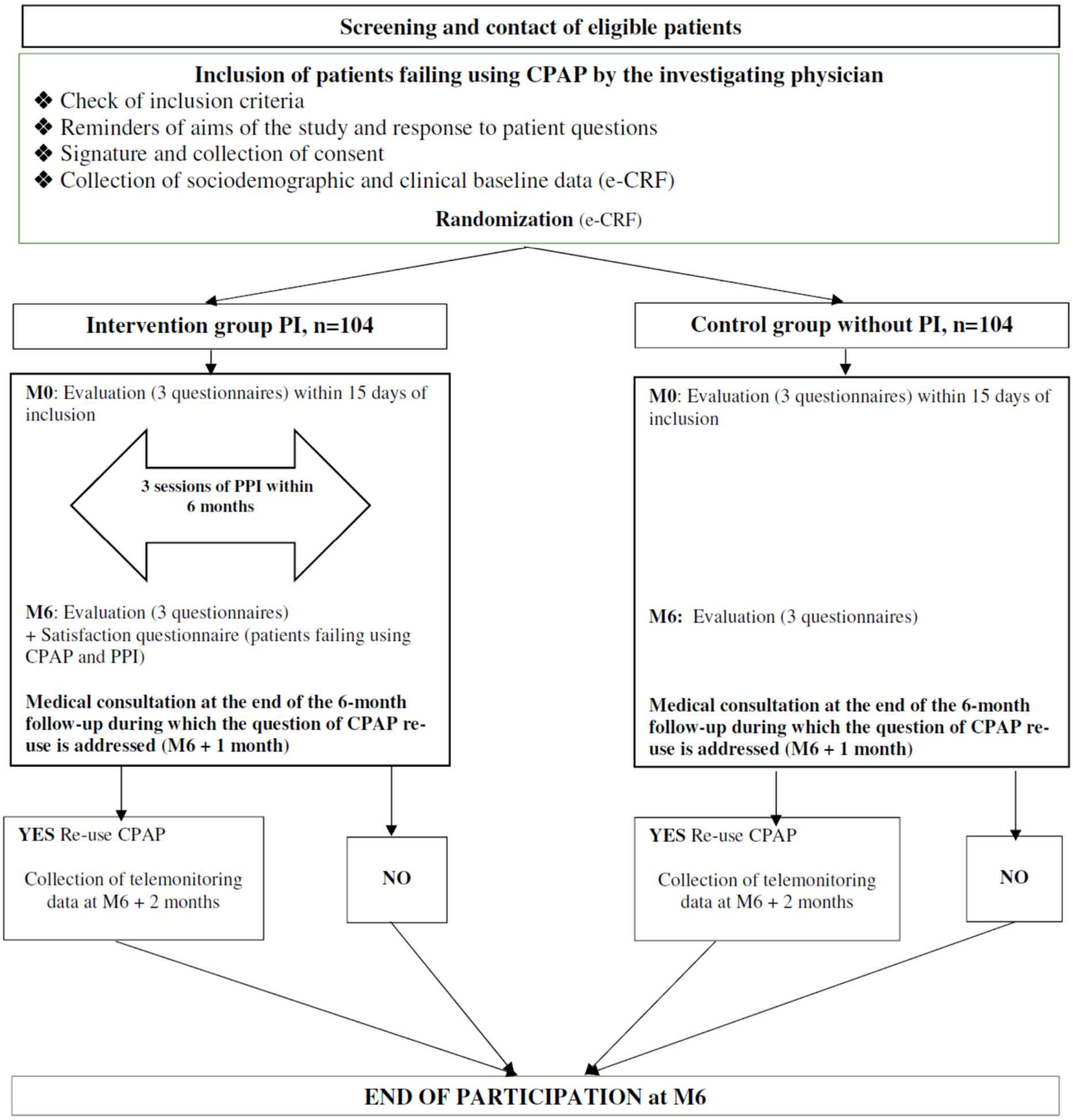

Supplementary file
SPIRIT 2013 Checklist

Section/item	ItemNo, ligne manuscrit	Description
**Administrative information**		
Title	1, 1-2	Descriptive title identifying the study design, population, interventions, and, if applicable, trial acronym
Trial registration	2a, 62	Trial identifier and registry name. If not yet registered, name of intended registry
	2b,	All items from the World Health Organization Trial Registration Data Set
Protocol version	3, joined, 6-05-2021, v1.1	Date and version identifier
Funding	4, 284-6	Sources and types of financial, material, and other support
Roles and responsibilities	5a, 340-9	Names, affiliations, and roles of protocol contributors
	5b, C. Pison, cpison@chu-grenoble.fr	Name and contact information for the trial sponsor
	5c, none	Role of study sponsor and funders, if any, in study design; collection, management, analysis, and interpretation of data; writing of the report; and the decision to submit the report for publication, including whether they will have ultimate authority over any of these activities
	5d, PI as C. Pison	Composition, roles, and responsibilities of the coordinating centre, steering committee, endpoint adjudication committee, data management team, and other individuals or groups overseeing the trial, if applicable (see Item 21a for data monitoring committee)
**Introduction**		
Background and rationale	6a, 86-124	Description of research question and justification for undertaking the trial, including summary of relevant studies (published and unpublished) examining benefits and harms for each intervention
	6b	Explanation for choice of comparators
Objectives	7, 135-149	Specific objectives or hypotheses
Trial design	8, Fig. 1	Description of trial design including type of trial (eg, parallel group, crossover,

For peer review only

31-05-2021 version, *BMJ open Protocol-R2, revised document, the 4th of September 2021*

		factorial, single group), allocation ratio, and framework (eg, superiority, equivalence, noninferiority, exploratory)
**Methods: Participants, interventions, and outcomes**		
Study setting	9, 152-5	Description of study settings (eg, community clinic, academic hospital) and list of countries where data will be collected. Reference to where list of study sites can be obtained
Eligibility criteria	10, Table 1	Inclusion and exclusion criteria for participants. If applicable, eligibility criteria for study centres and individuals who will perform the interventions (eg, surgeons, psychotherapists)
Interventions	11a, 157-191	Interventions for each group with sufficient detail to allow replication, including how and when they will be administered
11b, NA		Criteria for discontinuing or modifying allocated interventions for a given trial participant (eg, drug dose change in response to harms, participant request, or improving/worsening disease)
11c, Fig. 1		Strategies to improve adherence to intervention protocols, and any procedures for monitoring adherence (eg, drug tablet return, laboratory tests)
11d, none		Relevant concomitant care and interventions that are permitted or prohibited during the trial
Outcomes	12, 136-149	Primary, secondary, and other outcomes, including the specific measurement variable (eg, systolic blood pressure), analysis metric (eg, change from baseline, final value, time to event), method of aggregation (eg, median, proportion), and time point for each outcome. Explanation of the clinical relevance of chosen efficacy and harm outcomes is strongly recommended
Participant timeline	13, Fig. 1	Time schedule of enrolment, interventions (including any

For peer review only - http://bmjopen.bmj.com/site/about/guidelines.xhtml

		run-ins and washouts), assessments, and visits for participants. A schematic diagram is highly recommended (see Figure)
Sample size	14, 220-8	Estimated number of participants needed to achieve study objectives and how it was determined, including clinical and statistical assumptions supporting any sample size calculations
Recruitment	15, 229-33	Strategies for achieving adequate participant enrolment to reach target sample size
**Methods: Assignment of interventions (for controlled trials)**		
Allocation:		
Sequence generation	16a, 235-6	Method of generating the allocation sequence (eg, computer-generated random numbers), and list of any factors for stratification. To reduce predictability of a random sequence, details of any planned restriction (eg, blocking) should be provided in a separate document that is unavailable to those who enrol participants or assign interventions
Allocation concealment mechanism	16b, 235-6	Mechanism of implementing the allocation sequence (eg, central telephone; sequentially numbered, opaque, sealed envelopes), describing any steps to conceal the sequence until interventions are assigned
Implementation	16c, 235-6	Who will generate the allocation sequence, who will enrol participants, and who will assign participants to interventions
Blinding (masking)	17a, NA except outcome assessors	Who will be blinded after assignment to interventions (eg, trial participants, care providers, outcome assessors, data analysts), and how
17b, NA		If blinded, circumstances under which unblinding is permissible, and procedure for

For peer review only

		revealing a participant's allocated intervention during the trial
**Methods: Data collection, management, and analysis**		
Data collection methods	18a	Plans for assessment and collection of outcome, baseline, and other trial data, including any related processes to promote data quality (eg, duplicate measurements, training of assessors) and a description of study instruments (eg, questionnaires, laboratory tests) along with their reliability and validity, if known. Reference to where data collection forms can be found, if not in the protocol
	18b, 229-233	Plans to promote participant retention and complete follow-up, including list of any outcome data to be collected for participants who discontinue or deviate from intervention protocols
Data management	19, see Protocol	Plans for data entry, coding, security, and storage, including any related processes to promote data quality (eg, double data entry; range checks for data values). Reference to where details of data management procedures can be found, if not in the protocol
Statistical methods	20a, 237-265	Statistical methods for analysing primary and secondary outcomes. Reference to where other details of the statistical analysis plan can be found, if not in the protocol
	20b, NA	Methods for any additional analyses (eg, subgroup and adjusted analyses)
	20c, NA	Definition of analysis population relating to protocol non-adherence (eg, as randomised analysis), and any statistical methods to handle missing data (eg, multiple imputation)
**Methods: Monitoring**		
Data monitoring	21a, monitoring independant from investigators	Composition of data monitoring committee (DMC); summary of its role and reporting structure; statement of whether it is

For peer review only

independent from the sponsor and competing interests; and reference to where further details about its charter can be found, if not in the protocol. Alternatively, an explanation of why a DMC is not needed

5

For peer review only

31-05-2021 version, *BMJ open Protocol-R2, revised document, the 4th of September 2021*

21b, NA		Description of any interim analyses and stopping guidelines, including who will have access to these interim results and make the final decision to terminate the trial
Harms, NA	22	Plans for collecting, assessing, reporting, and managing solicited and spontaneously reported adverse events and other unintended effects of trial interventions or trial conduct
Auditing	23 every 3 months	Frequency and procedures for auditing trial conduct, if any, and whether the process will be independent from investigators and the sponsor
**Ethics and dissemination**		
Research ethics approval	24, 267-271	Plans for seeking research ethics committee/institutional review board (REC/IRB) approval
Protocol amendments	25, investigators	Plans for communicating important protocol modifications (eg, changes to eligibility criteria, outcomes, analyses) to relevant parties (eg, investigators, REC/IRBs, trial participants, trial registries, journals, regulators)
Consent or assent	26a, patient's doctor	Who will obtain informed consent or assent from potential trial participants or authorised surrogates, and how (see Item 32)
26b, NA		Additional consent provisions for collection and use of participant data and biological specimens in ancillary studies, if applicable
Confidentiality	27,	How personal information about potential and enrolled participants will be collected, shared, and maintained in order to protect confidentiality before, during, and after the trial
Declaration of interests	28, 359-61	Financial and other competing interests for principal investigators for the overall trial and each study site
Access to data	29, investigators	Statement of who will have access to the final trial dataset, and disclosure of

For peer review only

		contractual agreements that limit such access for investigators
Ancillary and post-trial care	30, NA	Provisions, if any, for ancillary and post-trial care, and for compensation to those who suffer harm from trial participation
Dissemination policy	31a, 279-82	Plans for investigators and sponsor to communicate trial results to participants, healthcare professionals, the public, and other relevant groups (eg, via publication, reporting in results databases, or other data sharing arrangements), including any publication restrictions
31b		Authorship eligibility guidelines and any intended use of professional writers
31c		Plans, if any, for granting public access to the full protocol, participant-level dataset, and statistical code

6

**Appendices**		
Informed consent materials	32, protocol	Model consent form and other related documentation given to participants and authorised surrogates
Biological specimens	33, NA	Plans for collection, laboratory evaluation, and storage of biological specimens for genetic or molecular analysis in the current trial and for future use in ancillary studies, if applicable

7

8

For peer review only

For peer review only

For peer review only - http://bmjopen.bmj.com/site/about/guidelines.xhtml

For peer review only

For peer review only - http://bmjopen.bmj.com/site/about/guidelines.xhtml

31-05-2021 version, *BMJ open Protocol-R1, revised document, the 4th of August 2021*

SPIRIT 2013 Checklist

**Section/item**	**ItemNo, ligne manuscrit**	**Description**
**Administrative information**		
Title	1, 1-2	Descriptive title identifying the study design, population, interventions, and, if applicable, trial acronym
Trial registration	2a, 62	Trial identifier and registry name. If not yet registered, name of intended registry
2b,		All items from the World Health Organization Trial Registration Data Set
Protocol version	3, joined, 6-05-2021, v1.1	Date and version identifier
Funding	4, 284-6	Sources and types of financial, material, and other support
Roles and responsibilities	5a, 340-9	Names, affiliations, and roles of protocol contributors
5b, C. Pison, cpison@chu-grenoble.fr		Name and contact information for the trial sponsor
5c, none		Role of study sponsor and funders, if any, in study design; collection, management, analysis, and interpretation of data; writing of the report; and the decision to submit the report for publication, including whether they will have ultimate authority over any of these activities
5d, PI as C. Pison		Composition, roles, and responsibilities of the coordinating centre, steering committee, endpoint adjudication committee, data management team, and other individuals or groups overseeing the trial, if applicable (see Item 21a for data monitoring committee)
**Introduction**		
Background and rationale	6a, 86-124	Description of research question and justification for undertaking the trial, including summary of relevant studies (published and unpublished) examining benefits and harms for each intervention
6b		Explanation for choice of comparators
Objectives	7, 135-149	Specific objectives or hypotheses
Trial design	8, Fig. 1	Description of trial design including type of trial (eg, parallel group, crossover, factorial, single group), allocation ratio, and framework (eg, superiority,

For peer review only

31-05-2021 version, *BMJ open Protocol-R1, revised document, the 4th of August 2021*

			equivalence, noninferiority, exploratory)
**Methods: Participants, interventions, and outcomes**			
Study setting	9, 152-5		Description of study settings (eg, community clinic, academic hospital) and list of countries where data will be collected. Reference to where list of study sites can be obtained
Eligibility criteria	10, Table 1		Inclusion and exclusion criteria for participants. If applicable, eligibility criteria for study centres and individuals who will perform the interventions (eg, surgeons, psychotherapists)
Interventions	11a, 157-191		Interventions for each group with sufficient detail to allow replication, including how and when they will be administered
11b, NA		Criteria for discontinuing or modifying allocated interventions for a given trial participant (eg, drug dose change in response to harms, participant request, or improving/worsening disease)	
11c, Fig. 1		Strategies to improve adherence to intervention protocols, and any procedures for monitoring adherence (eg, drug tablet return, laboratory tests)	
11d, none		Relevant concomitant care and interventions that are permitted or prohibited during the trial	
Outcomes	12, 136-149		Primary, secondary, and other outcomes, including the specific measurement variable (eg, systolic blood pressure), analysis metric (eg, change from baseline, final value, time to event), method of aggregation (eg, median, proportion), and time point for each outcome. Explanation of the clinical relevance of chosen efficacy and harm outcomes is strongly recommended
Participant timeline	13, Fig. 1		Time schedule of enrolment, interventions (including any run-ins and washouts), assessments, and visits for participants. A schematic

For peer review only - http://bmjopen.bmj.com/site/about/guidelines.xhtml

31-05-2021 version, *BMJ open Protocol-R1, revised document, the 4th of August 2021*

		diagram is highly recommended (see Figure)
Sample size	14, 220-8	Estimated number of participants needed to achieve study objectives and how it was determined, including clinical and statistical assumptions supporting any sample size calculations
Recruitment	15, 229-33	Strategies for achieving adequate participant enrolment to reach target sample size
**Methods: Assignment of interventions (for controlled trials)**		
Allocation:		
Sequence generation	16a, 235-6	Method of generating the allocation sequence (eg, computer-generated random numbers), and list of any factors for stratification. To reduce predictability of a random sequence, details of any planned restriction (eg, blocking) should be provided in a separate document that is unavailable to those who enrol participants or assign interventions
Allocation concealment mechanism	16b, 235-6	Mechanism of implementing the allocation sequence (eg, central telephone; sequentially numbered, opaque, sealed envelopes), describing any steps to conceal the sequence until interventions are assigned
Implementation	16c, 235-6	Who will generate the allocation sequence, who will enrol participants, and who will assign participants to interventions
Blinding (masking)	17a, NA except outcome assessors	Who will be blinded after assignment to interventions (eg, trial participants, care providers, outcome assessors, data analysts), and how
17b, NA		If blinded, circumstances under which unblinding is permissible, and procedure for revealing a participant's allocated intervention during the trial
**Methods: Data collection, management, and analysis**		

For peer review only

31-05-2021 version, *BMJ open Protocol-R1, revised document, the 4th of August 2021*

Data collection methods	18a	Plans for assessment and collection of outcome, baseline, and other trial data, including any related processes to promote data quality (eg, duplicate measurements, training of assessors) and a description of study instruments (eg, questionnaires, laboratory tests) along with their reliability and validity, if known. Reference to where data collection forms can be found, if not in the protocol
	18b, 229-233	Plans to promote participant retention and complete follow-up, including list of any outcome data to be collected for participants who discontinue or deviate from intervention protocols
Data management	19, see Protocol	Plans for data entry, coding, security, and storage, including any related processes to promote data quality (eg, double data entry; range checks for data values). Reference to where details of data management procedures can be found, if not in the protocol
Statistical methods	20a, 237-265	Statistical methods for analysing primary and secondary outcomes. Reference to where other details of the statistical analysis plan can be found, if not in the protocol
	20b, NA	Methods for any additional analyses (eg, subgroup and adjusted analyses)
	20c, NA	Definition of analysis population relating to protocol non-adherence (eg, as randomised analysis), and any statistical methods to handle missing data (eg, multiple imputation)
**Methods: Monitoring**		
Data monitoring	21a, monitoring independant from investigators	Composition of data monitoring committee (DMC); summary of its role and reporting structure; statement of whether it is independent from the sponsor and competing interests; and reference to where further

For peer review only

details about its charter can be found, if not in the protocol. Alternatively, an explanation of why a DMC is not needed

3

For peer review only

31-05-2021 version, *BMJ open Protocol-R1, revised document, the 4th of August 2021*

21b, NA		Description of any interim analyses and stopping guidelines, including who will have access to these interim results and make the final decision to terminate the trial
Harms, NA	22	Plans for collecting, assessing, reporting, and managing solicited and spontaneously reported adverse events and other unintended effects of trial interventions or trial conduct
Auditing	23 every 3 months	Frequency and procedures for auditing trial conduct, if any, and whether the process will be independent from investigators and the sponsor
**Ethics and dissemination**		
Research ethics approval	24, 267-271	Plans for seeking research ethics committee/institutional review board (REC/IRB) approval
Protocol amendments	25, investigators	Plans for communicating important protocol modifications (eg, changes to eligibility criteria, outcomes, analyses) to relevant parties (eg, investigators, REC/IRBs, trial participants, trial registries, journals, regulators)
Consent or assent	26a, patient's doctor	Who will obtain informed consent or assent from potential trial participants or authorised surrogates, and how (see Item 32)
26b, NA		Additional consent provisions for collection and use of participant data and biological specimens in ancillary studies, if applicable
Confidentiality	27,	How personal information about potential and enrolled participants will be collected, shared, and maintained in order to protect confidentiality before, during, and after the trial
Declaration of interests	28, 359-61	Financial and other competing interests for principal investigators for the overall trial and each study site
Access to data	29, investigators	Statement of who will have access to the final trial dataset, and disclosure of

For peer review only

		contractual agreements that limit such access for investigators
Ancillary and post-trial care	30, NA	Provisions, if any, for ancillary and post-trial care, and for compensation to those who suffer harm from trial participation
Dissemination policy	31a, 279-82	Plans for investigators and sponsor to communicate trial results to participants, healthcare professionals, the public, and other relevant groups (eg, via publication, reporting in results databases, or other data sharing arrangements), including any publication restrictions
	31b	Authorship eligibility guidelines and any intended use of professional writers
	31c	Plans, if any, for granting public access to the full protocol, participant-level dataset, and statistical code

Appendices

Informed consent materials	32, protocol	Model consent form and other related documentation given to participants and authorised surrogates
Biological specimens	33, NA	Plans for collection, laboratory evaluation, and storage of biological specimens for genetic or molecular analysis in the current trial and for future use in ancillary studies, if applicable

For peer review only
